# Beyond the heterodimer model for mineralocorticoid and glucocorticoid receptor interactions in nuclei and at DNA

**DOI:** 10.1371/journal.pone.0227520

**Published:** 2020-01-10

**Authors:** John R. Pooley, Caroline A. Rivers, Michael T. Kilcooley, Susana N. Paul, Ayse Derya Cavga, Yvonne M. Kershaw, Serena Muratcioglu, Attila Gursoy, Ozlem Keskin, Stafford L. Lightman

**Affiliations:** 1 Henry Wellcome Laboratories for Integrative Neuroscience and Endocrinology, University of Bristol, Bristol, United Kingdom; 2 Laboratory of Receptor Biology and Gene Expression, National Cancer Institute, National Institutes of Health, Bethesda, Maryland, United States of America; 3 Department of Chemical and Biological Engineering, Koc University, Istanbul, Turkey; 4 Department of Molecular and Cell Biology, University of California, Berkeley, California, United States of America; 5 Howard Hughes Medical Institute, University of California, Berkeley, California, United States of America; Université de Genève, SWITZERLAND

## Abstract

Glucocorticoid (GR) and mineralocorticoid receptors (MR) are believed to classically bind DNA as homodimers or MR-GR heterodimers to influence gene regulation in response to pulsatile basal or stress-evoked glucocorticoid secretion. Pulsed corticosterone presentation reveals MR and GR co-occupy DNA only at the peaks of glucocorticoid oscillations, allowing interaction. GR DNA occupancy was pulsatile, while MR DNA occupancy was prolonged through the inter-pulse interval. In mouse mammary 3617 cells MR-GR interacted in the nucleus and at a chromatin-associated DNA binding site. Interactions occurred irrespective of ligand type and receptors formed complexes of higher order than heterodimers. We also detected MR-GR interactions *ex-vivo* in rat hippocampus. An expanded range of MR-GR interactions predicts structural allostery allowing a variety of transcriptional outcomes and is applicable to the multiple tissue types that co-express both receptors in the same cells whether activated by the same or different hormones.

## Introduction

Endogenous glucocorticoid hormones (cortisol and corticosterone) are synthesized and secreted in response to stressful life experiences as well as released under basal conditions with well-defined basal circadian and ultradian rhythms [1, 2]. It is well known that stress-evoked glucocorticoid release yields transcriptional responses [3], but the ultradian rhythm additionally generates a pulsatile transactivation or transrepression of gene targets that maintains basal gene- and cell-type specific transcriptional programs [4–6]. This pulsatile gene activation does not occur in response to the commonly used synthetic glucocorticoids, which especially during long term use, are associated with metabolic and neuropsychiatric side-effects [7]. A better understanding of the dynamic aspects glucocorticoid signalling pathways is crucial for the rational design of better therapeutic agents.

Glucocorticoids act through two related steroid hormone receptors, the glucocorticoid receptor (GR) and the mineralocorticoid receptor (MR). The MR has approximately a 10-fold higher affinity for corticosterone than GR and produces different activation patterns in response to ultradian and stress-induced stimulation [8, 9]. These ligand-activated transcription factors share a common domain structure [10, 11] with a ligand binding domain (LBD) harbouring the ligand binding pocket and an activation function (AF2) domain. A short hinge region separates the LBD from the DNA binding domain (DBD) that contains two zinc fingers providing somewhat flexible recognition of the DNA sequence 5’-TGTTCT-3’, and allowing the binding of an inverse palindromic glucocorticoid response element (GRE) separated by three base pairs (5’-GGAACAnnnTGTTCT-3’) as a dimer [12]. MR/GR undergo nuclear translocation following hormone binding and possess unique amino terminal domains (NTD) incorporating the activation function domain (AF1).

The LBD and DBD between MR and GR are highly similar at the structural and sequence level, reflecting similar hormone selectivity [13] and identical DNA binding site recognition [14]. By interpreting of models of the isolated DBD, both are understood to dimerize through the D-loop in the second zinc finger [12, 15–19], however molecular studies have supported flexibility in steroid receptor interactions. For example, ER and TR/RXR interact in different ways using in some cases, alternative interfaces [12, 20–23]. The established dimer model, together with the 94% DBD sequence identity between MR/GR [24], lead to the expectation that MR/GR could interact through heterodimerization [25, 26]. The MR-GR complex was anticipated to take the same form as the DNA-bound GR homodimer [12], replacing GR on one half of the palindromic sequence with MR, then conferring a gene regulatory role distinct from GR or MR alone, though the exact functional outcome may be dependent on the promoter or cell line [27]. Synergy at transient reporter constructs [26], but also inhibition of GR-mediated responses [25, 28] has been documented, while MR-GR complexes can be more effective transrepressors than either receptor alone [29]. Such work established MR-GR heterodimerization as a mechanism extending the regulatory potential of glucocorticoids in co-expressing cell types [26].

Although MR-GR interaction at DNA oligomers *in vitro* has been well demonstrated [25, 26, 29–31], few efforts have been made to extend these observations to living cells or tissue [9, 32, 33]. Herein the context of chromatin provides considerably greater restriction to receptor DNA access, even for two proteins recognising the same DNA motif [34]. Moreover, additional dimerization surfaces for MR and GR have been proposed [35, 36] and it is now understood that the DNA sequence and surrounding proteins likely confer additional levels of regulation to receptor structure and interaction potential through inter-domain, allosteric communication [37–39].

In brain regions such as the hippocampus and parvocellular paraventricular nucleus (PVN), as well as adipocytes, osteoblasts, immune cell types and kidney, where MR and GR are co-expressed within the same cells [40–44], MR/GR are afforded ample opportunity to cooperatively regulate gene expression and physiology. For instance, full GR-mediated suppression of evening ACTH requires low level corticosterone occupying MR [45]. In CA1 neurons both receptors are required for corticosterone regulation of low threshold inactivating and high threshold non-inactivating calcium conductance and the I_Q_ current [46, 47].

Supporting experimental investigations with structural modelling of crystallised domains, we investigated the potential for MR-GR to interact in the living cell. Our data strongly support interaction in the nucleus and at a chromatinised DNA template, but additionally indicate a previously unrecognised level of complexity. MR-GR may interact through a variety of surfaces allowing the formation of a range MR-GR complexes beyond simple heterodimers. In conflict with the structural heterodimer previously proposed, receptor mutagenesis did not reveal an extensive role for the D-loop in cells. We report MR-GR interaction independently of the type of bound ligand (agonist, antagonist) and at low corticosterone concentrations associated with the pulse peaks of the ultradian rhythm where both receptors co-load at DNA. The heterodimerization model for MR-GR interaction appears incomplete and our findings considerably expand the potential modes by which MR and GR can cooperate to regulate gene expression in conjunction with other allosteric mediators.

## Results

### MR and GR interact in cell line and hippocampus lysates

**Fig 1:**
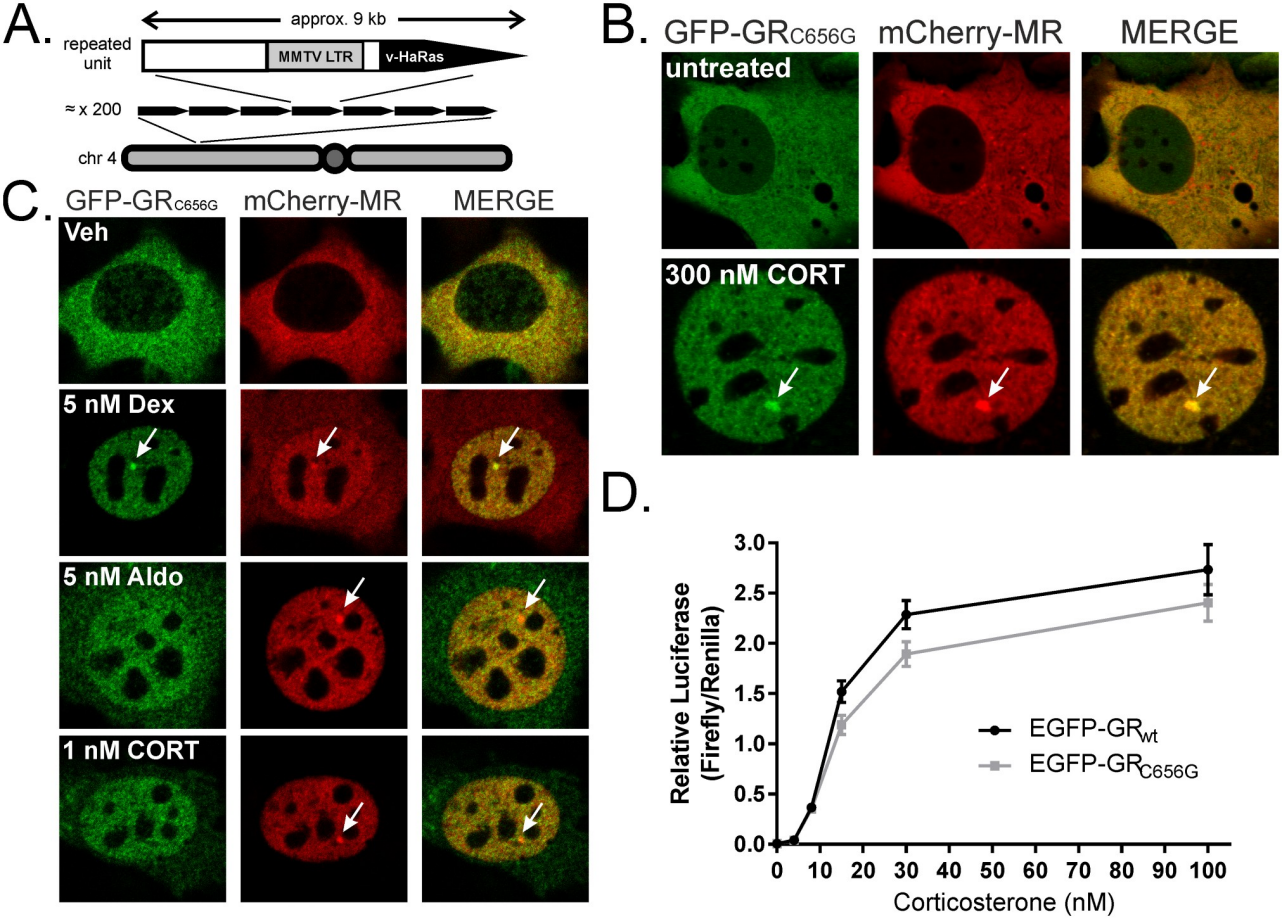
A cell line model for MR and GR co-expression. (A) The repeating unit in the MMTV array comprises the MMTV LTR driving expression of viral Harvey Ras (HaRas) cDNA. Mammary adenocarcinoma line 3617 contains approximately 200 copies at one location in chromosome 4. (B) 3617ChMR cells express fluorescently tagged receptors that localize to the cytoplasm in the absence of hormone but undergo nuclear translocation and bind the array structure (arrows) in the presence of corticosterone. (C) Dexamethasone (5 nM) induces array loading of both receptors, complete GFP-GR_C656G_ translocation, but partial mCherry-MR translocation. Aldosterone (5 nM) induces mCherry-MR array loading, complete mCherry-MR translocation, but partial GFP-GR_C656G_ translocation. 1 nM corticosterone favours mCherry-MR translocation, likely due MR’s higher affinity for corticosterone relative to GR. Treatments were 30 min. (D) The GR_C656G_ mutation has little effect on the corticosterone sensitivity of EGFP-GR. COS-1 cells containing no endogenous GR or MR were transfected with equivalent amounts of wildtype (wt) or C656G mutant rat EGFP-GR prior to treatment with corticosterone for 24 hrs at the doses indicated. Co-transfected pFC31-Luc provided a MMTV-driven firefly luciferase reporter and pRL-CMV a Renilla transfection control. Two-way ANOVA of Renilla-corrected luminescence showed a significant effect of dose (F(5,24) = 190.4, p<0.001), and of mutation (F(1,24) = 7.54, p = 0.011), but no interaction (dose × mutation, F(5,24) = 1.43, p = 0.251). The effect of mutation was minimal, independent samples t-testing produced no significant differences between wild type and mutant receptor output at any dose (p = 0.625, 0.717, 0.702, 0.086, 0.103, and 0.350 for 0, 4, 8, 15, 30, and 100 nM respectively). Mean ± SEM, n = 3.

Murine 3617 cells contain a chromatinised array of head-to-tail copies of the MMTV long-terminal repeat (LTR) providing 800–1200 GREs in close proximity (Fig 1A). MR or GR binding at this site is observable as focal receptor accumulation within the surrounding nucleoplasm [48]. A tetracycline repressed GFP-tagged rat GR_C656G_ responds to corticosterone or dexamethasone in 3617 [4]. Retroviral addition of tetracycline repressed mCherry-tagged MR produced a cell line (3617ChMR) expressing both MR and GR, mirroring co-expression in hippocampal neurons [40]. Both receptors are cytoplasmic in the absence of hormone but translocate into the nucleus and load at the MMTV array after corticosterone treatment (Fig 1B). More selective ligands (dexamethasone at GR, aldosterone at MR) provoked preferential translocation of the appropriate receptor (Fig 1C). Incorporated GR_C656G_ produces higher affinity for dexamethasone [49] but luciferase assays demonstrated little effect on corticosterone sensitivity (Fig 1D). Therefore, 1 nM corticosterone permitted complete mCherry-MR, but incomplete GFP-GR_C656G_ translocation (Fig 1C).

**Fig 2:**
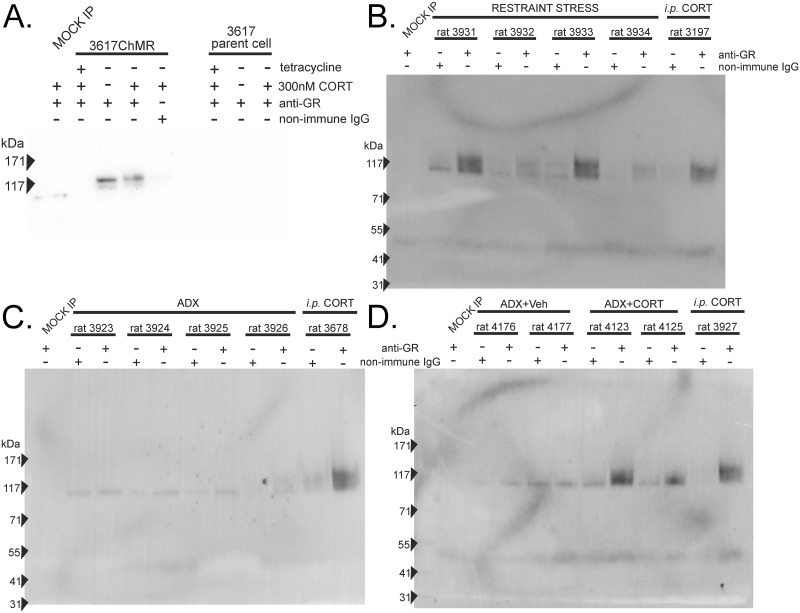
Co-immunoprecipitation supports MR-GR interaction in a cell line and whole rat hippocampus. (A) Anti-GR immunoprecipitation additionally pulls down mCherry-MR when 3617ChMR cells are treated with vehicle or corticosterone. Negative controls include a non-immune IgG as the immunoprecipitating antibody and a MOCK immunoprecipitation containing antibody and buffer but no lysate. Parent 3617 cells do not express mCherry-MR but contain GFP-GR and endogenous mouse GR. mCherry-MR expected at 136 kDa. (B) Immunoprecipitation of rat GR from whole hippocampus additionally captures MR supporting interaction following stress exposure. Mean plasma corticosterone for these animals was 366 ng/ml (range 326–434 ng/ml). Endogenous MR expected at 107 kDa. (C) MR does not co-immunoprecipitate with GR when animals are bilaterally adrenalectomized (ADX, mean corticosterone 17 ng/ml, range 6–36 ng/ml). Compare signal to positive control (i.p. CORT) animal. (D) Intraperitoneal corticosterone injection (i.p., 3 mg/kg) of ADX rats restores MR co-immunoprecipitation indicating hormone dependence. Mean corticosterone was 617 ng/ml (range 516–717 ng/ml) when killed 30 min after injection at the corticosterone peak [8]. Animals for i.p. CORT controls were adrenally intact and injected with 3 mg/kg corticosterone 30 min before death (mean plasma corticosterone 586 ng/ml, range 375–948 ng/ml). Smeared MR bands with the anti-MR 1D5 antibody may suggest MR post-translational modifications. Faint bands around 50 kDa represent the non-specific labelling of the immunoprecipitating IgG common to many co-IPs.

Biochemical work has demonstrated MR and GR can interact as a functional transcription complex proposed, but not directly demonstrated, to be a heterodimer. Confirming biochemical support for MR-GR interaction, an anti-GR antibody recognizing both endogenous and GFP-tagged GR co-immunoprecipitated mCherry-MR from 3617ChMR lysates (Fig 2A). Co-immunoprecipitation was independent of hormone treatment but occurred only if tetracycline repression was released allowing both GFP-GR_C656G_ and mCherry-MR to express. The western antibody was not cross-reactive to GR as no co-immunoprecipitated MR was obtained from 3617 parent cells containing only GFP-GR_C656G_ and endogenous mouse GR.

We next examined rat hippocampus where MR/GR co-express endogenously. Whole hippocampus lysates co-immunoprecipitated MR with GR when rats had undergone 30 min restraint stress before sacrifice (Fig 2B). Variation in the extent of the signal and background level was observed, but specific antibody consistently produced bands exceeding the intensity of a non-immune IgG control for the same lysate. This was not the case for adrenalectomized animals where signal was similar between IgG and anti-GR, but co-immunoprecipitation of MR could be restored by prior intraperitoneal injection with corticosterone (Fig 2C and 2D). Thus, in the hippocampus, MR-GR interactions appear corticosterone dependent and occur during the normal physiological response to stress.

### Ultradian pulse peaks permit MR-GR interactions at DNA

**Fig 3:**
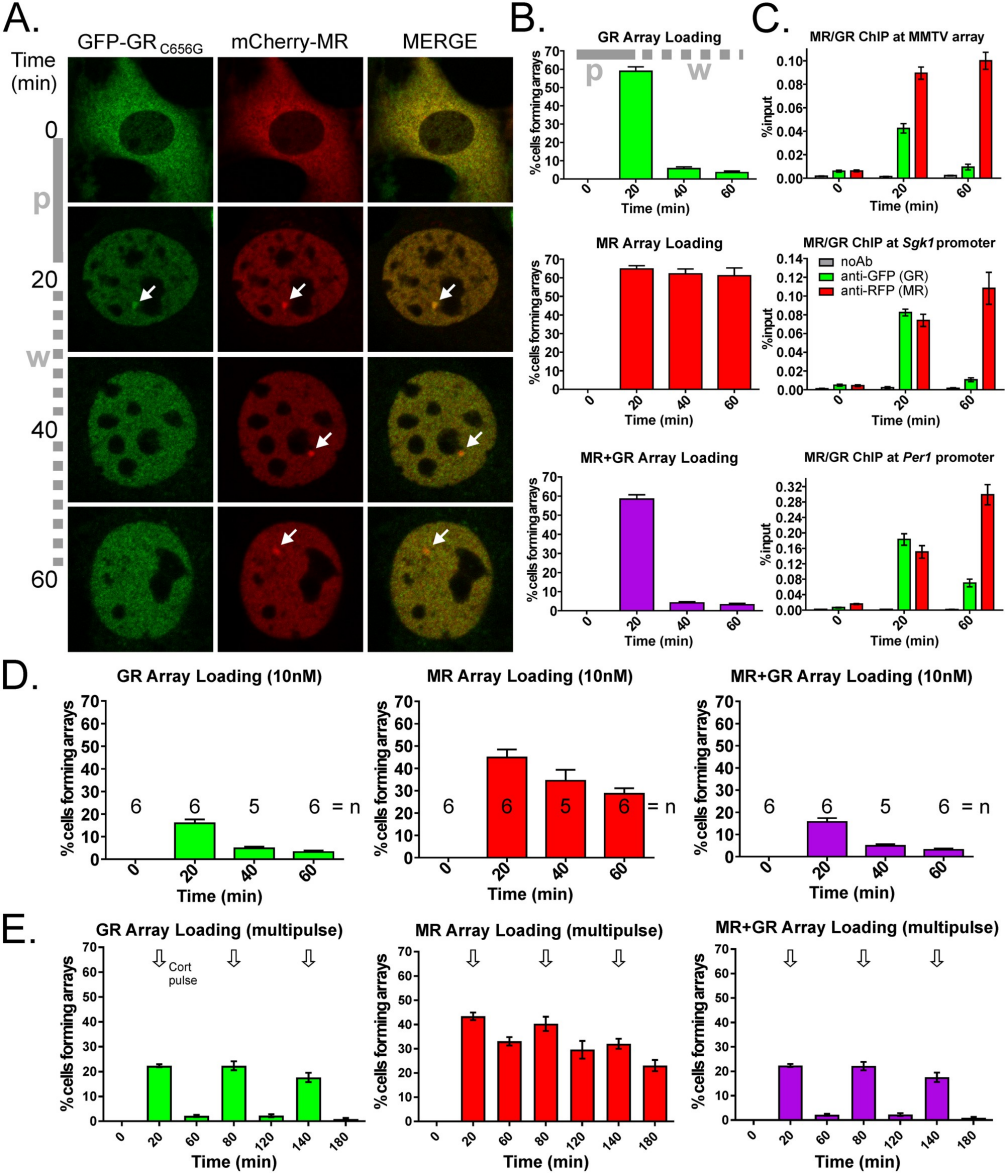
MR and GR binding during simulated ultradian corticosterone pulsatility. (A) Representative 3617ChMR cells following 20 min, 100 nM corticosterone pulse (p) + washout (w). MMTV array formation (arrows) provides measurable loading of fluorescently tagged GR and MR at chromatinised DNA. (B) Percentage total cells over multiple fields of view forming arrays loading GFP-GR_C656G_, mCherry-MR, or both receptors on the same array. Mean ± SEM, n = 6 from two independent experiments, ≥130 cells per replicate. Counting 0 min samples was not routinely performed (cytoplasmic receptors) but a baseline of 2–3% cells formed weak intranuclear arrays in the absence of corticosterone. (C) ChIP targeting fluorescent receptor tags. Transient EGFP-GR_C656G_ and prolonged mCherry-MR loading were observed at the MMTV array structure and at GRE-containing endogenous gene promoters *Sgk1* and *Per1*. Mean ± SEM, n = 3. (D) Percentage of total cells forming arrays loading GFP-GR_C656G_ and/or mCherry-MR. An ultradian pulse in the physiological range was simulated with 10 nM corticosterone. Mean ± SEM from two independent experiments. (E) Three successive 20 min pulses of 30 nM were applied 1 hr apart to mimic the ultradian rhythm (arrows). Each pulse produced a transient increase in GFP-GR_C656G_ array loading, while mCherry-MR array loading was largely unresponsive to ultradian stimulation. The downward drift in occupancy over the time course likely relates to accumulated media changes. Mean ± SEM, n = 6 from two independent experiments.

Interaction between MR and GR has long been expected in conditions where glucocorticoid levels are high during the stress response (Fig 2B). It is less clear whether interactions occur at lower hormone levels associated with the endogenous ultradian rhythm. We next examined the DNA binding characteristics of fluorescently tagged MR and GR_C656G_ over a simulated ultradian pulse of corticosterone in 3617ChMR cells to determine MR/GR co-loading at DNA over the ultradian cycle. Cells were presented with a 20 min pulse of 100 nM corticosterone, after which two media changes removed hormone from the cells (termed ‘washout’, simulating the falling phase of an ultradian pulse). Such corticosterone presentation previously allowed cyclical GR DNA binding to drive gene pulsing at endogenous genes [4]. Ethanol vehicle alone caused no receptor movement (S1 Fig), while at the peak of the corticosterone pulse, mCherry-MR and GFP-GR_C656G_ had translocated and bound to the MMTV array (Fig 3A and 3B). Over the washout period GFP-GR_C656G_ DNA binding reduced considerably while the level of mCherry-MR association remained high. Maintained mCherry-MR and transient GFP-GR_C656G_ array binding was verified by ChIP targeting receptor fluorescent tags. Prolonged mCherry-MR but transient EGF-GR_C656G_ binding was observed at endogenous GREs in the *Sgk1* and *Per1* promoters (Fig 3C). When either MR or GR bind chromatinised DNA receptors undergo continuous turnover on a timescale of seconds. Initially demonstrated for GR using fluorescence recovery after photobleaching (FRAP) at the MMTV array [48], this ‘hit and run’ model for receptor action is additionally applicable to MR using the same approach (S1 Fig).

To reproduce the concentrations of free corticosterone measured by microdialysis in the hippocampus *in vivo* [50, 51], we reduced the corticosterone concentration to 10 nM (a relatively high free corticosterone ultradian pulse in female Wistar rats). The total numbers of arrays observed to bind GFP-GR_C656G_ was lower at 10 nM although DNA binding remained strictly associated with the hormone pulse peak (Fig 3D). Conversely, mCherry-MR loaded at the pulse peak and was largely maintained at the end of the washout (≥60% retention at 60 min). Though at this dose mCherry-MR had some capacity to reduce its levels of DNA occupancy, it required longer than the inter-pulse interval to come off DNA entirely (more than 120 min with 5 nM corticosterone, S1 Fig).

To test the possibility that mCherry-MR retains DNA occupancy in the 60 min inter-pulse period directly, 3617ChMR were treated with three successive pulse-washouts of 30 nM corticosterone ensuring arrays loaded sufficient GFP-GR_C656G_ to be detectable. A majority of mCherry-MR remained loaded at the MMTV array between pulses, while GFP-GR_C656G_ continued to be cyclically associated with the array at the pulse peaks (Fig 3E). Together, these data imply that any MR-GR interactions at DNA during the ultradian rhythm would be limited to the pulse peaks when both receptors load at binding sites.

### MR and GR interact in the nucleoplasm and at DNA within living cells

**Fig 4:**
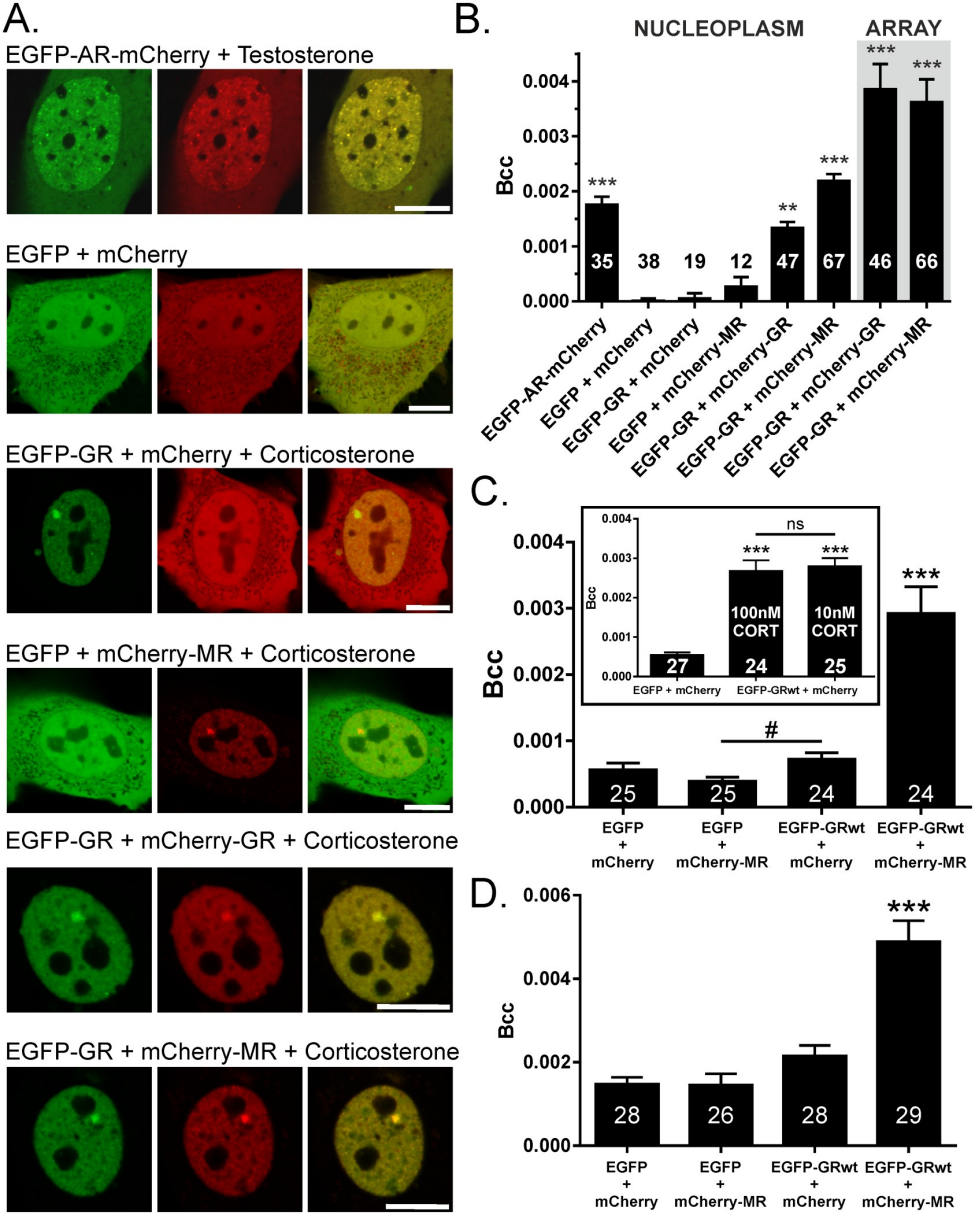
Cross-correlation number and brightness assay (ccN&B) reveals MR-GR interactions in the nucleoplasm of 3617 cells and at chromatin. (A) Representative cells (scale bars 10 μm). 3617 cells +tetracycline transfected with constructs expressing the proteins indicated 24 hrs before imaging. Use of GR_C656G_ mirrored co-immunoprecipitation experiments. Nuclear localisation was driven by at least 30 min treatment with 1 μM testosterone (EGFP-AR-mCherry) or 300 nM corticosterone (all other conditions). (B) An androgen receptor (AR) tagged at one end with EGFP and at the other with mCherry provided a positive interaction control. AR does not bind the MMTV array but nucleoplasmic Bcc values were significantly higher than negative controls EGFP + mCherry, EGFP-GR_C656G_ + mCherry, and EGFP + mCherry-MR. Bcc values were additionally obtained from GR+GR and MR+GR pairings in nucleoplasm and at the MMTV array. (C) Interaction of mCherry-MR and EGFP-GR_wt_ at 100 nM corticosterone in 3617 nucleoplasm. Bcc values between groups were significantly different (F(3,48.17) = 14.57, p < 0.001). Inset compares Bcc values obtained using 100 nM or 10 nM corticosterone, group means were significantly different (F(2,35.6) = 77.46, p < 0.001). There was no significant difference (ns) between groups with different corticosterone doses. (D) Neuronal cell line N2a shows significant MR-GR interaction in the nucleoplasm relative to negative controls. Group means were significantly different (F(3,56.44) = 14.95, p < 0.001). EGFP_A207K_ prevents EGFP dimerization at higher concentrations. Welch’s F test for main effects with Games-Howell post hoc testing. Means ± SEM, number of cells indicated within or over the bar. Means significantly different from all negative control group(s) where **p < 0.01, ***p < 0.001. # Significantly different from each other, p = 0.023.

Given the presence of both receptors on the same arrays during corticosterone presentation we next sought to determine the extent of interaction at physiologically relevant loci within the cell. Cross-correlation number and brightness assay (ccN&B) examined interactions of separately tagged fluorescent proteins by correlating their intensity fluctuations in time similar to fluorescence cross-correlation spectroscopy [52, 53]. Correlated intensity fluctuations are reflected in the Bcc value which is positive when interactions occur, and was obtained in corticosterone treated, transiently transfected, 3617 cells pairing MR and GR_C656G_ (as for 3617ChMR cells), and for additional positive and negative controls (Fig 4A and 4B). Welch’s F test revealed a significant effect of transfection, F(7,93.42) = 81.79, p < 0.001. Games-Howell post hoc testing indicated the MR+GR_C656G_ pairing showed a significantly higher Bcc value in the nucleoplasm relative to EGFP + mCherry, EGFP + mCherry-MR, or EGFP-GR_C656G_ + mCherry negative controls (each p < 0.001), supporting interaction in live cells. Crucially MR-GR_C656G_ interaction was also observed at the MMTV array, a focal accumulation of DNA-bound receptors (p < 0.001 relative to each negative control). As the Bcc value contains information relating to both the amount of interaction occurring and the stoichiometry of the interacting complexes, interpretation of higher Bcc values at arrays relative to nucleoplasm is challenging.

Wild type rat GR was also found to interact with MR in 3617 nucleoplasm at 100 nM corticosterone, and at a lower dose of 10 nM corticosterone consistent with ultradian pulse peaks (Fig 4C). Similar studies on N2a cells revealed MR-GR interactions in the nucleoplasm of a corticosterone treated neuronal cell type (Fig 4D). Although the MR+GR Bcc value was still significant (p < 0.01 relative to all other groups) without modification of the protocol, an EGFP mutation preventing dimerization at higher concentrations was used for this cell type (A207K in our EGFP) [54].

Taken together these data suggest MR and GR interact in the nucleoplasm of living cells and at a chromatinised DNA template, supporting the expectation that these complexes have transcriptional roles.

### Intracellular assessment does not support utilization of the D-loop to mediate MR-GR interaction or a large heterodimer population

**Fig 5:**
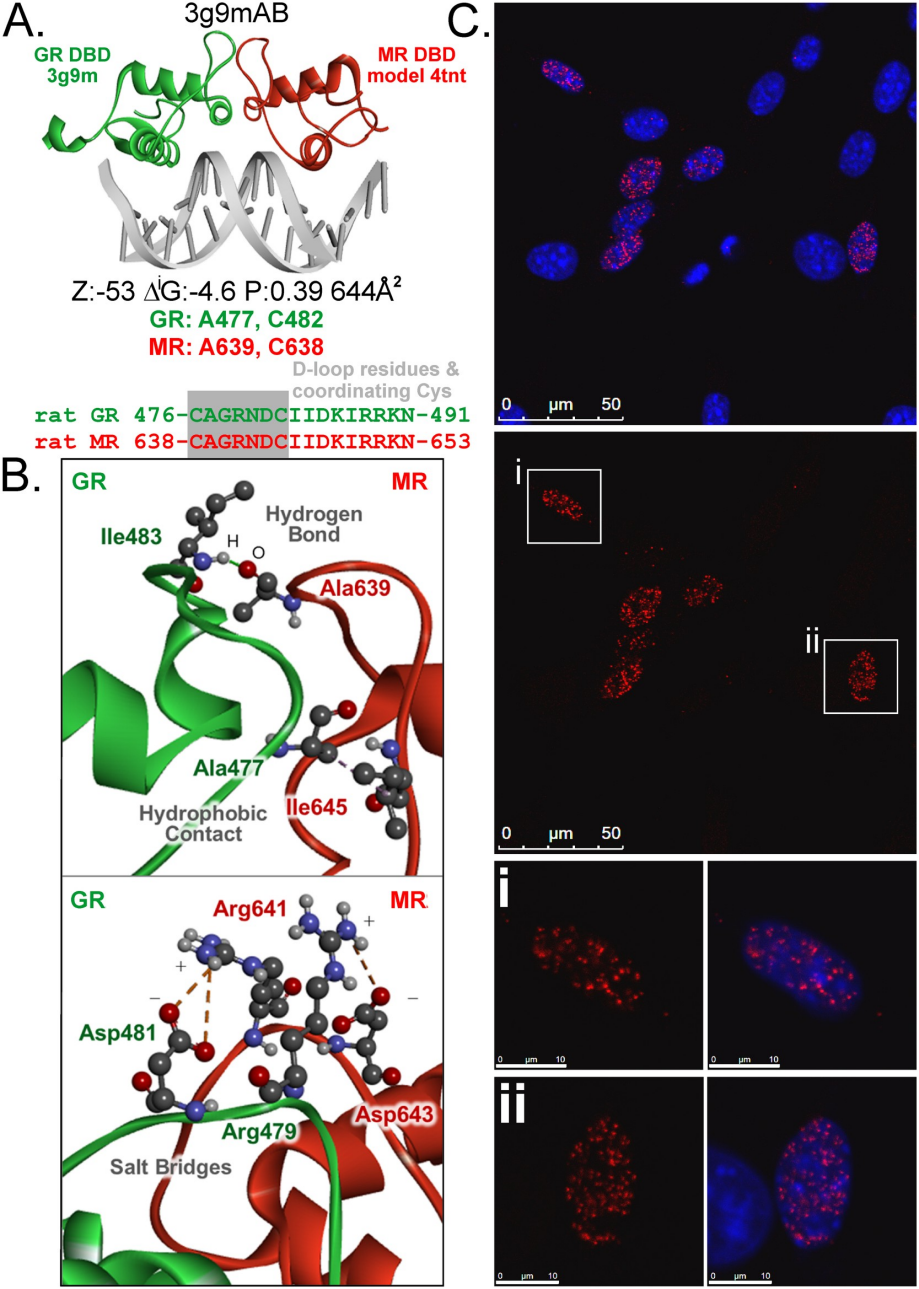
Using proximity ligation assay to detect interaction between MR and GR. (A) Structural prediction for MR and GR interaction through the classical dimerization domain in the D-loop of the DNA binding domain. Linear protein sequence highlighting D-loop amino acids using rat amino acid numbering for GR (green) and MR (red). The PDB ID for the rat GR DBD is 3G9MA. Rat MR DBD is a homology model based on human MR DBD (PDB ID 4TNTA). The interaction interface template matched by PRISM is 3g9mAB, which is a rat GR DBD homodimer. The energy score predicted by ZRANK is -53, the solvation free energy predicted by PDBePISA Δ^i^G is -4.6 kcal/mol, interface area is 644Å^2^, and the Δ^i^G P-value is 0.39, which passes the significance threshold of 0.5. Interacting hot spots on GR and MR are provided below the structure with complete hot spot and interface residues in S1 Table. (B) Representation of selected D-loop interface contacts of the second zinc finger mediating the interaction in (A). (C) Proximity ligation assay (PLA) identifies MR-GR interaction in 3617 nuclei transfected with untagged receptors and corticosterone treated (100 nM) for 45 min. Nuclei stained with DAPI (blue) while red dots are focal amplifications of ligated DNA probe marking interaction sites. Scale bar is 50 μm (large panels), or 10 μm (lower panels/individual cells).

**Fig 6:**
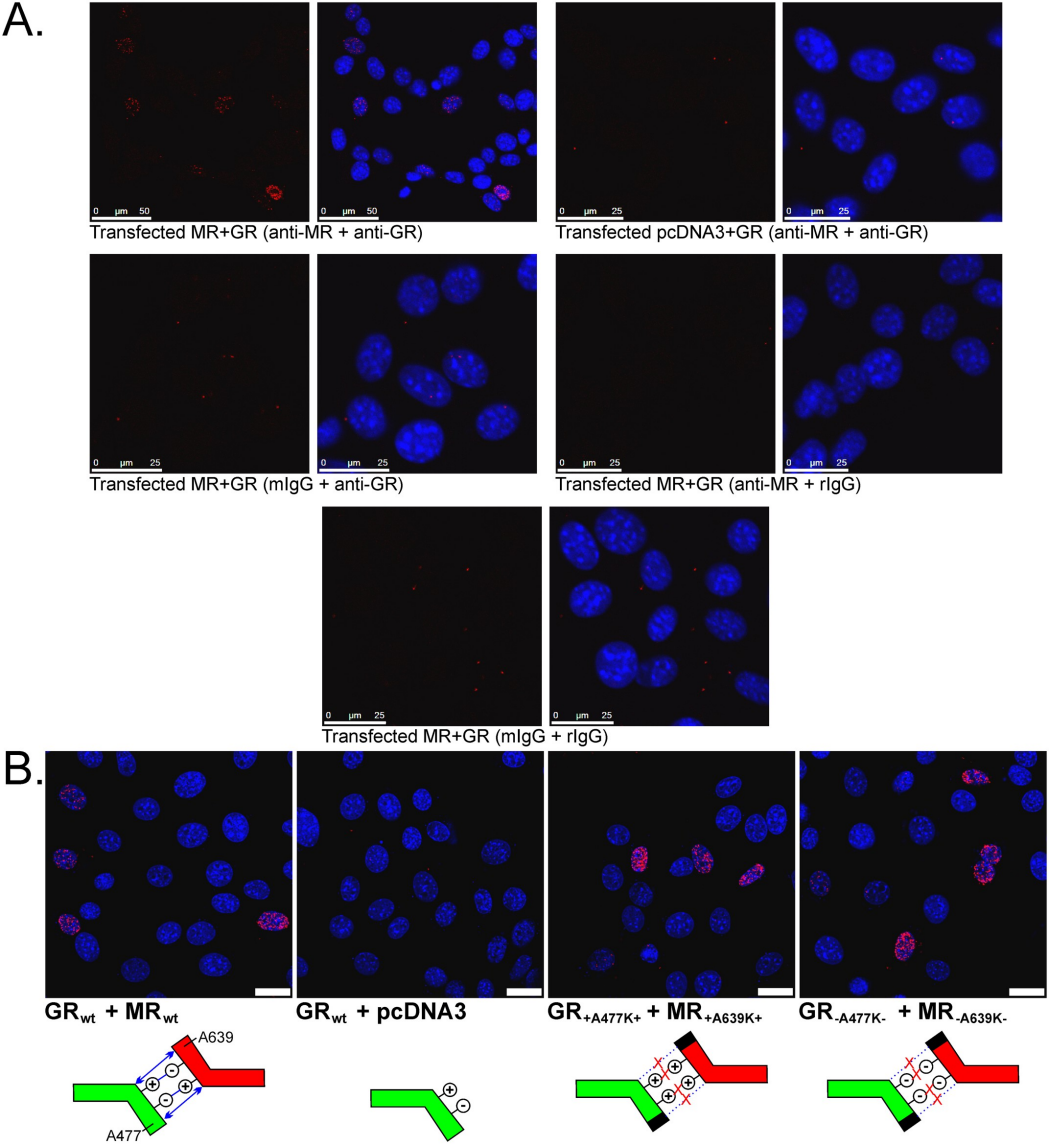
MR-GR interaction in 3617 nucleoplasm does not require the classical heterodimerization interface in the D-loop. (A) Positive PLA signal is obtained only when both receptors and specific antibodies are present. Non-specific background was assessed by replacing primary antibodies with non-immune IgG from the appropriate species. Scale bar = 25 μm aside from the first panel (50 μm). (B) Mutations in the D-loop targeting the expected heterodimer interface do not prevent MR-GR interaction. Schematics show the extent to which A477K (GR)/A639K (MR) mutations combined with mutations in the salt bridge disrupt the expected interface. For GR_+A477K+_ and MR_+A639K+_ constructs the salt bridge changes made were D481R and D643R (GR and MR respectively). For GR_-A477K-_ and MR_-A639K-_ constructs R479D (GR) and R641D (MR) mutations were made. Scale bars = 25 μm.

The structural heterodimer proposed by others constitutes a DNA bound dyad of MR and GR DNA binding domains (DBD) interacting through an interface in the D-loop of the second zinc finger (Fig 5A and 5B). Computational modelling confirmed this theoretical association of DBDs underpinning the heterodimer model (Fig 5A, S1 Table). To directly test the utilisation of this interface to mediate MR-GR interaction in cells, we combined mutations in the D-loop with the proximity ligation assay (PLA), for which one pairing of antibodies produced amplicons through rolling circle replication labelling MR-GR interactions in transfected 3617 nuclei supporting ccN&B data (Fig 5C). PLA was validated in 3617 cells using appropriate negative controls (Fig 6A) and cross-reactivity between antibodies was ruled out with immunohistochemistry targeting transfected receptors in 3617_M20-_ cells, which were CRISPR-engineered to remove the anti-GR M-20 epitope from endogenous mouse GR (S2 Fig). Structural modelling of the proposed heterodimer interface allowed calculation of mutations expected to be disruptive. Due to its high pair potential and average ΔΔG score the A477K change in GR was expected to be highly disruptive without needing to alter the coordinating cysteines in the zinc finger (S2 Table). This residue, previously targeted for the GRdim change A477T, was also a hot spot in MR. Although calculations did not suggest that mutations of residues forming the asymmetric salt bridge contacts would be as disruptive, these have nonetheless been consistently targeted by others examining MR-GR interaction [25, 28, 55]. We elected to make both changes at equivalent residues in MR and GR and test the effect of mutagenesis on MR-GR interactions by PLA. To avoid potential interference from endogenous mouse GR we used 3617_M20-_ cells in which the epitope for the anti-GR antibody had been deleted (these cells do not express MR endogenously). PLA signal was readily observed for wildtype and mutated receptors despite the disruption caused to the D-loop, suggesting that this interface was not required to support at least a majority of the MR-GR interactions in 3617 nuclei detected by PLA (Fig 6B). The interaction may be mediated by the ligand binding domain.

To investigate the nature of the MR-GR interaction in 3617 cells we used the number and brightness assay [56] to compare the average molecular brightness (ε) of fluorescent receptors in the nucleus of 3617 cells paired with different co-expression partners by virtue of measuring the variance in fluorescence intensity of the moving population at each pixel within the nucleoplasm. In principle the average molecular brightness doubles with each doubling of molecular stoichiometry (the number of particles within the complex), but because the ε value is an average of the population, mixed populations are not well handled by the approach and a 1:1 mixture of trimers and monomers has the same ε value as a population of dimers. The data of others also advises caution in overinterpretation of this data [57], but it holds that higher order complexes are associated with higher ε values.

**Fig 7:**
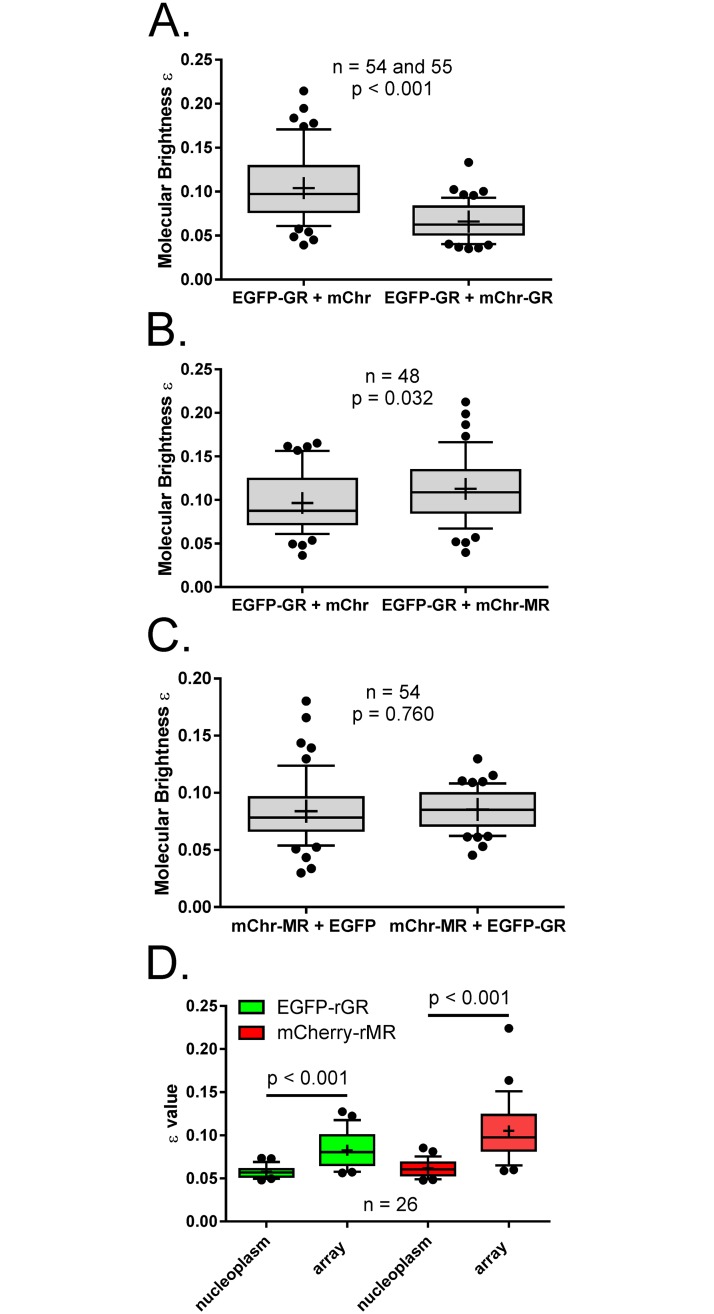
Experimental evidence suggests higher order MR-GR complexes in 3617. (A) Number and brightness assay (N&B) from the green channel only to compare EGFP-rGR average molecular brightness in the presence of mCherry or mCherry-rGR. The ε value of EGFP-rGR reduces in the presence of mCherry-rGR consistent with lower stoichiometry and heterodimer formation. (B) The ε value recorded from the green channel (EGFP-rGR) in the presence of mCherry-rMR is slightly but significantly higher than in the presence of mCherry alone. (C) The ε value for mCherry-rMR in the red channel is not significantly different when co-expressed with EGFP or EGFP-rGR. (D) The molecular brightness (stoichiometry) for both EGFP-rGR and mCherry-MR in co-transfected 3617 cells was significantly higher at the MMTV array than in the surrounding nucleoplasm. Measurements were obtained from the channel in which the box plot is coloured (green, EGFP-rGR; red, mCherry-rMR). Independent samples t-tests, equal variances not assumed. All cells measured had corticosterone added at 100 nM (A-C) or 300 nM (D). Data shows median (line), mean (+) and the 25-75^th^ percentiles at the box boundaries. Whiskers are the 10-90^th^ percentiles with outliers as black dots.

EGFP-rGR was paired with mCherry with which it does not interact (Fig 4B), or with mCherry-rGR with which it was expected to form a population of differentially labelled green-red heterodimers in addition to green-green and red-red homodimers. Measuring only the green channel (EGFP-rGR), red molecules are invisible, therefore green-red dimers appear to be monomers and the average ε value significantly reduces when EGFP-rGR is paired with mCherry-rGR (Fig 7A). The magnitude of this decrease was mathematically consistent with differentially labelled red-green heterodimers forming with equal probabilities of assembly as green-green homodimers (ε value projected at 66.67% of the +mCherry reference, observed 63.3%), supporting previous reports that GR is found as a dimer in the nucleoplasm of corticosterone treated 3617 cells [58]. A similar decrease in ε when EGFP-rGR was measured paired with mCherry-rMR was anticipated from *in vitro* work anticipating preferential MR-GR heterodimer formation over homodimers [26], but this was not the case and a slight increase in ε was evident (Fig 7B). The lack of decreased brightness suggests GR-MR interactions do not occur, by enlarge, as 1:1 heterodimers. The molecular brightness of mCherry-rMR was additionally consistent in the presence of EGFP or EGFP-rGR (Fig 7C). As N&B reports the population average molecular brightness we could not rule out a small population of MR-GR heterodimers assembling at DNA undetectable in nucleoplasm. We therefore measured ε at the MMTV array and compared these values to those obtained from the nucleoplasm in the same cells. Heterodimer formation specifically at MMTV DNA would be expected to report lower ε values at the array compared to those in the nucleoplasm but the molecular brightness was higher at the array for both EGFP-rGR and mCherry-MR (Fig 7D) suggesting the presence of higher oligomeric states. Together with PLA data these results are not consistent with the interaction of MR and GR as a true 1:1 heterodimer in living cells but suggest multimers not reliant on the D-loop dimerization interface.

### DBD and LBD structural modelling supports multiple alternative interfaces allowing MR-GR interaction

**Fig 8:**
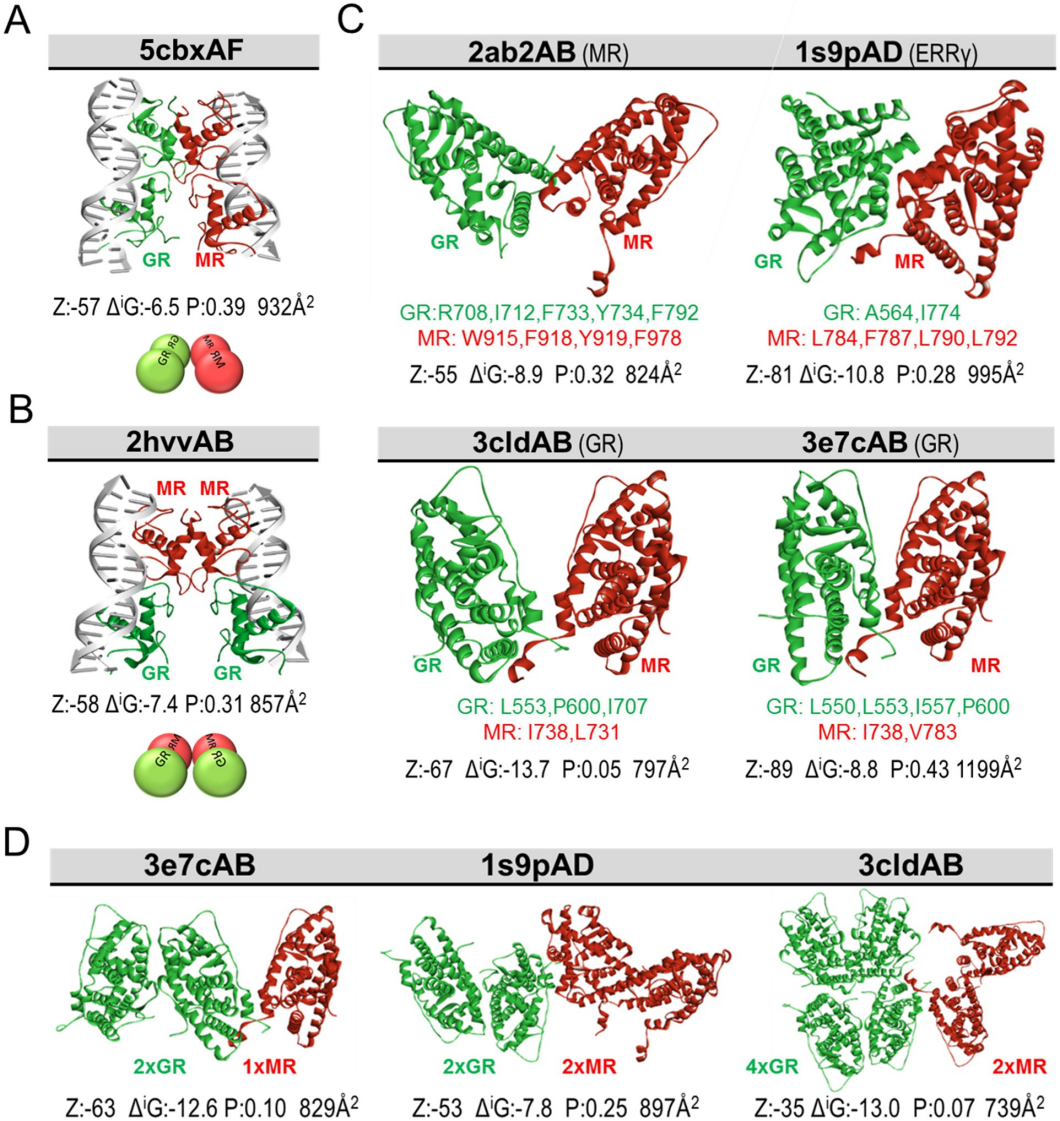
Structural predictions for MR-GR interactions through the DBD or LBD. The interaction interface template is indicated above each structure. The following information is provided below each model; interaction energy scores predicted by ZRANK (Z), interface area in Å^2^, the solvation free energy Δ^i^G and the Δ^i^G P-value predicted by PDBePISA. Each model is below the significance threshold of 0.5 P-value, indicating high hydrophobicity and significance according to PDBePISA. Each interface also has energy levels comparable to the template interfaces from PDB (S4 Table). (A) A possible mode of DBD-mediated interaction of a GR+GR homodimer with a MR+MR homodimer. Structure could theoretically occur by looping intervening DNA between palindromes. (B) A possible mode of DBD-mediated interaction between two MR-GR heterodimers forming a tetramer at DNA, potentially looping out DNA between binding sites. The DBD structures from PDB are 1R4R and 3G6P for GR, and a rat homology model of 4TNT for MR. Complete hot spot and interface residues for parts A and B provided in S3 Table. (C) MR and GR LBDs may associate through multiple alternative interfaces from nuclear receptor family crystal structures indicated in parentheses. GR LBD structures used are rat models based on 4p6x (cortisol) and MR structures are rat models based on 2aa2 (aldosterone). Residues provided below each structure are interacting hot spots and/or those with highest pair potential. Complete hot spot and interface residues are provided in S5 Table. (D) Assortment of predicted assemblies through the LBD illustrating the range of structural complexes and various interfaces available for MR and GR to interact.

To predict how MR and GR might interact, we used available crystal structures of these receptors together with the PRISM: Protein Interactions by Structural Matching tool [59, 60] version 2. Interaction models predicted by PRISM were dominated by template interfaces from the nuclear receptor family crystal structures as expected. Our N&B data for MR-GR suggested a higher order complex at the MMTV array compared to the nucleoplasm consistent with recent data for GR [61]. Consequently, we explored structural possibilities for higher order stoichiometries revealing alternative modes by which MR-GR could theoretically multimerise through their DBDs. A GR homodimer loaded at one GRE and a MR homodimer or monomer loaded at a second GRE could potentially loop to form tetrameric complexes (Fig 8A, S3 Table). Similarly, heterodimers loaded at separate GREs could also interact (Fig 8B, S3 Table).

Full length receptors include several additional domains in addition to the DBD, of which the ligand binding domain (LBD) has been crystallised. Computational predictions revealed multiple alternative candidate LBD interfaces for interactions between MR and GR (Fig 8C, S5 Table). The availability of alternative interfaces offers diversity to MR-GR interaction, and a variety of potential molecular complexes containing in some cases multiples of each receptor. Complexes predicted included anticipated heterodimers (1× MR, 1× GR, Fig 8C), but also a range of other possible arrangements from a dimer of dimers (2× GR, 2× MR) to 4× GR with 2× MR (Fig 8D). Our computational predictions with both the DBD and LBD imply that MR and GR might interact in a variety of different ways considerably expanding the heterodimer model and consistent with our experimental data revealing 1:1 heterodimer formation is not a dominant arrangement in 3617 cells.

**Fig 9:**
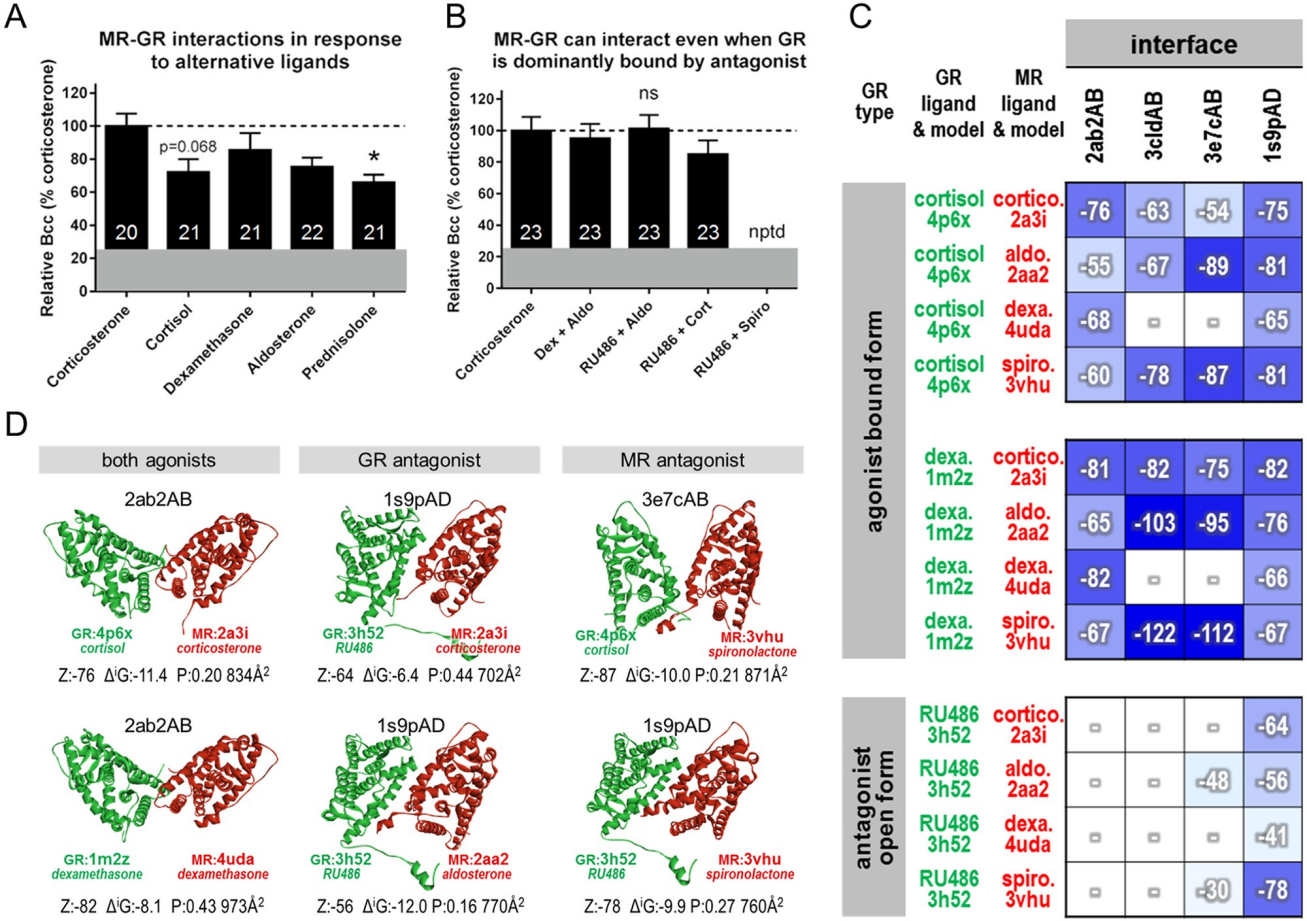
MR and GR can interact independently of the ligands in the receptor binding pockets. (A) Experimental comparison of Bcc values determined by ccN&B for MR-GR where different endogenous or synthetic ligands have been applied to activate both receptors (100 nM). The Bcc value for corticosterone (known interaction) was set to 100%. The greyed-out region represents the maximum mean Bcc value obtained for non-interacting controls in previous experiments. (B) Experimentally determined Bcc values for combined treatments expressed relative to 100 nM corticosterone (known interaction). Doses were varied in accordance with expectations around ligand selectivity to obtain a likely dominant occupation of each receptor by the appropriate ligands. Dexamethasone 10 nM + aldosterone 10 nM, spironolactone + RU486 (1 μM each), aldosterone + RU486 and corticosterone + RU486 (10 nM MR-targeted agonist, 1 μM GR-targeted antagonist). Mean ± SEM, number of cells shown within bar. *Significantly different, p<0.05; ns, not significant; nptd, not possible to determine. (C) ZRANK binding energy scores of computational predictions of alternative interfaces between MR and GR. Different agonist and antagonist combinations and structural conformations demonstrate the potential for multiple modes of interaction through the LBD regardless of the bound ligand, with varying interface preference. Note the different trends in interface preferences, especially with the open antagonist bound form. cortico., corticosterone; aldo, aldosterone; dexa, dexamethasone; spiro, spironolactone; “–”indicates no predictions were found by PRISM for the indicated pair of proteins through the template interface. (D) Structural examples of MR-GR interactions through the LBD when both are agonist bound, and when GR or MR is antagonist bound. For each pair of interactors, the most favourable interface is displayed with the PDB IDs of the templates for rat homology modelling provided below each structure. The following information is provided below each model; interaction energy scores predicted by ZRANK (Z), interface area in Å^2^, the solvation free energy Δ^i^G and the Δ^i^G P-value predicted by PDBePISA.

We tested the capacity for full length MR-GR to interact in 3617 cells when treated with 100 nM of alternative endogenous and synthetic ligands using ccN&B (Fig 9A, S3 Fig). One-way ANOVA indicated a significant effect of ligand (F(4,100) = 2.94, p = 0.024), with post hoc testing showing only prednisolone produced a significantly lower Bcc value relative to corticosterone (p = 0.027), though a similar trend was observed for cortisol (p = 0.068). Similarly, we tested the ability of MR-GR to interact when each receptor was dominantly activated by alternative agonists, or where GR bound largely RU486 but MR largely aldosterone or corticosterone (Fig 9B, S3 Fig). One-way ANOVA reported that each agonist/antagonist pairing produced similar Bcc values to corticosterone alone (F(3,88) = 0.715, p = 0.546), though it was not possible to determine a nuclear Bcc value for the pairing of RU486 and spironolactone (minimal mCherry-MR translocation, insufficiently bright). Although the bound ligand generally did not affect the ability of MR-GR to interact in our structural computations or experimental work, the LBD interfaces utilised were computationally predicted to be influenced by the ligand, suggesting allosteric transfer of information from the ligand binding pocket to the MR-GR structural interaction. Different trends in interface preferences were observed when GR was agonist bound compared to when it was antagonist bound in an open form (Fig 9C and 9D). The open antagonist-bound form where the N-terminal end extending toward the DBD is outward (3h52 modelled structure, Fig 9D) has a much more limited interface usage.

## Discussion

We show MR and GR interact within the living cell nucleus and at DNA providing opportunity to mediate the transcriptional responses expected of such complexes [14, 26]. MR-GR interactions need not conform to the structural heterodimer previously theorised and we propose a range of possible interactions is likely, permitting explanation of the variety of data obtained for MR-GR interaction in different model systems, and broadening understanding of how MR/GR interact with the genome to promote transcriptional responses specific to cell type and DNA binding site. We propose allosteric flexibility through oligomerisation as a mechanism for alternative transcriptional outcomes at the gene regulatory level for MR-GR interaction as described for other signalling proteins [62], establishing a method by which two highly similar transcription factors can respond to the same endogenous hormones and bind the same DNA recognition sites, but deliver such diversity. This is an important consideration for understanding the molecular basis of the side effect profile of commonly used synthetic glucocorticoids.

### Interaction of MR and GR

Co-immunoprecipitation of MR with GR in 3617ChMR cells confirmed previous biochemical reports that MR and GR can interact [25, 26, 29, 31]. We extend this work to MR/GR co-expressing rat hippocampus [40] showing these receptors interact following exposure to stress or corticosterone (Fig 2). Utilizing two approaches (ccN&B and PLA) that rely on different principles we observed interactions in 3617 nuclei mirroring observations in COS1 cells [32]. As the Bcc measure contains information relating to both the frequency of interaction and the stoichiometry of the complex present, we do not consider it appropriate to significantly weight the magnitudes of the signals obtained, interpreting this data only as an interaction when the signal is higher than that obtained from negative control conditions.

Interactions were observed to be dependent on corticosterone availability in the rat hippocampus while independent of the presence of corticosterone in 3617 cells. It has previously been reported that nuclear receptor cytoplasmic interactions in the absence of ligand in cell lines may be an artefact [63]. Yet data implying the contrary also exist, suggesting the cytoplasmic interaction may be hormone independent in some cell types such as COS7 [33], but dependent in others such as COS1 [32] contingent on the milieu of MR/GR chaperones or post-translational modifying proteins available.

Connecting intranuclear interactions to chromatin and gene regulatory events has been challenging. Various authors observe MR-GR interaction at oligonucleotides containing a range of GREs [26, 29–31] but overlook a potential regulatory role for chromatin in which receptor access is considerably more restricted, with differential access for similar steroid hormone receptors binding the same sequence [34]. Elegant demonstration of MR/GR occupying the same strand of *Fkbp5* DNA at the same time in rat hippocampal chromatin was important [9], but MR/GR may be located at separate sites on the 400 bp average chromatin fragment size. Examining MR/GR at the MMTV array in 3617 cells we found significant cross-correlation signals consistent with direct evidence for MR-GR interaction at chromatinised DNA in the living cell (Fig 4). Together with ChIP studies demonstrating a DNA bound GR was required to recruit a DNA-binding-defective MR [64], the functional role of MR-GR interactions as a transcriptional regulator is now clear. Obtaining *in vivo* direct evidence for interaction using PLA is a logical next step in parallel to exploring which genes are regulated by such complexes. As a potential caveat to our ccN&B approach to measuring receptor interactions at DNA, we note that it is only possible to rule out MR/GR separately loading at nearby GREs within the array contributing to the Bcc data obtained provided that movement of any DNA locus containing both simultaneously bound receptors is considerably slower than receptor movements. We assume that such DNA movements are not detected (appear static because far slower than scanner head) on the basis that pixel dwell time and pixel size were optimised around receptor diffusion coefficients 1–2 orders of magnitude faster than those reported for individual chromatin loci [65, 66].

Nuclear hormone receptors can form mixed ligand dimers in solution [67] and the type of ligand modulates heterodimeric interactions for other nuclear receptors. RXR-TR heterodimerization is reduced when RXR binds *9-cis*-retinoic acid [68]. As MR and GR can bind to the same or different hormones (e.g. MR+aldosterone, GR+cortisol), ligand context required assessment. Structural predictions with the LBDs suggested MR and GR could interact while binding a variety of alternative ligands. Prednisolone and cortisol had 60 or 70% respectively the Bcc value of corticosterone, which may indicate a weaker level of interaction, reduced complex stoichiometry, or both. Dexamethasone and aldosterone however gave Bcc values similar to corticosterone strongly supporting interaction. When MR/GR were treated with a pair of selective agonists at doses encouraging receptor preference, or with low doses of MR agonists paired with excess GR antagonist, Bcc values continued to support interactions between MR-GR. Mixed ligand complexes may explain how trout MR suppresses GR2-mediated reporter transactivation when bound to cortisol, 11-deoxycorticosterone, or MR antagonist eplerenone [28].

The expression of 11β-hydroxysteroid dehydrogenase 2 in many peripheral tissues has led to the expectation that MR-GR interactions could not occur in such locations as endogenous glucocorticoids are prevented from binding MR. By showing that interactions can occur irrespective of the ligand, we pose new questions about the physiological relevance of such interactions beyond the brain in tissues where MR activity is dominated by aldosterone, and about the potential for exogenous ligands to influence MR-GR interactions. MR and GR co-expression has now been described in neurons of the hippocampus and paraventricular nucleus of the hypothalamus [40], adipocytes [42], kidney distal nephron [44], immune cells [43], and osteoblasts [41].

### Physiological opportunity for MR-GR interaction

GR [48] and MR (herein) occupy chromatinised DNA only briefly, cycling bound and unbound states in seconds. This ‘hit and run’ mechanism arises through the actions of receptor-recruited ATP-dependent remodelling proteins [69], prevents competition between factors binding the same recognition site [70], and allows multiple complexes to load at the same site through dynamic assisted loading [64, 71].

*In vivo* glucocorticoid release occurs approximately every 60 min [2]. MR loading at the MMTV array displayed longevity that persisted even in experiments using low dose ultradian pulses of corticosterone where only a ≤40% reduction in occupancy during the washout period was observed. Prolonged MR DNA binding was concordant with data showing hippocampal MR remains nuclear beyond the inter-pulse interval [8]. It is probable that the longevity of MR DNA occupancy relates, at least in part, to its 10-fold higher affinity for corticosterone than GR. This difference in affinity between MR and GR has become a fundamental feature of MR versus GR signaling permitting U-shaped dose responses in glucocorticoid-sensitive neurophysiology [72]. Our findings however also suggest more flexibility in MR DNA binding than implied by near continuous corticosterone occupancy [13]. Such a view is consistent with recent work [9] using ChIP assay which demonstrates MR increased DNA loading after stressor exposure despite what should already be a very high baseline occupancy of the MR by corticosterone. It is not clear why MR DNA occupancy and ligand fractional occupancy do not mirror each other but candidates to govern this dissociation have been proposed and warrant further interrogation [9]. Together these data appear consistent with MR being selectively responsive to glucocorticoid availability during stress and the circadian rhythm but minimally responsive to the ultradian rhythm as it is not clear what effect the small reduction of no more than 40% binding might have during washout.

Our ChIP studies at 100 nM corticosterone suggest increased MR loading following washout at endogenous genes, particularly *Per1*. This finding implied GR/MR competition but this is difficult to rationalise as receptors loading the same site do not compete due to rapid exchange [70]. It is possible that the MR epitope became more accessible to antibody recognition following the departure of GR from some binding sites and this accounts for the observed ChIP signal.

As previously demonstrated [4, 6], GR DNA binding is lost during the nadir of ultradian pulses leaving opportunity for MR-GR interaction only during the pulse peaks of the ultradian cycle when both receptors engage DNA. At lower doses of corticosterone (5–10 nM) we noted that only around 25–36% of cells producing arrays (calculated as (purple bar/red bar)*100) loaded both receptors at the same array, compared to 50% at 30 nM and 90% at 100 nM. As the GR_C656G_ historically integrated into 3617 derived cells has a comparable corticosterone sensitivity to the wild type receptor, these data are consistent with MR-GR interactions increasing with corticosterone availability [32], and perhaps on the nuclear availability of the lower affinity GR which does not fully translocate at 1-10nM within the 20 minute pulse time. Yet ccN&B indicated similar Bcc measurements for intranuclear MR-GR interaction at both 10 nM and 100 nM corticosterone and others have observed functional effects of interactions with cortisol in the low nanomolar range [26]. Overall, our data support the view that MR-GR interaction can occur at hormone levels in the ultradian range, however loss of hippocampal intranuclear GR between pulses [6] implies a new translocation cycle for each pulse that may limit the formation of MR-GR complexes *in vivo*. No shared MR/GR DNA occupancy was reported under basal (circadian/ultradian) conditions specifically at hippocampal gene *Fkbp5* [9]. MR-GR complexes may be discovered to play a gene- and cell-type specific role communicating signals from basal HPA axis activity, while current evidence supports their involvement in the genomic response to stressor exposure.

### Where is the MR-GR interface?

Crystallization of the GR DBD at DNA revealed a dimer [12] with an interface in the D-loop of the second zinc finger [18]. High conservation in this region between steroid hormone receptors implied heterodimerization [73] and these results were extrapolated to MR-GR, projecting that DBD of the GR would bind to one half of the inverted repeat while the DBD for MR would bind to the other (Fig 5A and 5B). We directly tested the importance of this interface for full length MR-GR interaction in 3617 nuclei using PLA with a mutagenesis approach in which the most disruptive structural prediction (GR A477K, MR A639K) was combined with disruption of the salt bridge consistently targeted for MR-GR [25, 28]. MR and GR still interacted (Fig 6B) supporting evidence that this region was not required for trout MR to inhibit transactivation through GR2 [28] and rodent MR need not bind DNA to augment GR-mediated actions at endogenous genes [64]. Using N-terminal truncated receptors, the D-loop was important for full expression of MR-mediated inhibition, though mutation far from eliminated the effect [25]. Similar outcomes have been observed for GR homodimers where the D-loop contributes at best slightly to the interaction in the living cell nucleus [58]. We now reveal parallel findings indicating that the D-loop does not govern interaction between many MR and GR molecules, suggesting both limits of the D-loop mediated heterodimer model, but also that there must be alternatives. As both the number of cells showing PLA activity and the number of dots per cell are each as dependent on transfection efficiency in this approach as on the amount of interaction the number of dots visible per cell is subject to additional variables.

We predict alternative interfaces that could mediate MR-GR interaction in these contexts, finding multiple possible arrangements for both DBDs and LBDs. We do not rule out further interfaces within the intrinsically disordered NTD and hinge of these molecules. These predictions appear to explain the range of experimental data with MR-GR interactions mediated through the DBDs [25, 74], the MR NTD [31], and the GR LBD [33]. Conversely, studies have also expressly ruled out the interface of MR-GR being located in the NTD [26, 33], the DBD D-loop [28], the hinge [33], or the LBD [31]. Such conflicting information could relate to the use of receptors from different species but may reflect a potential for context-dependent utilization of interfaces. Previously, such findings have been interpreted as a multi-faceted dimerization interface centred on the D-loop with support from other regions. Instead we propose multiple possible MR-GR arrangements of which at least a majority in 3617 cells are not assembled through the D-loop. Like others [28], and consistent with conflicting reports of the interface location, we argue that MR and GR may interact in multiple modes parallel to other nuclear receptors. It is not yet clear whether multiple MR-GR complexes can exist within the same cell type, or whether different complexes confer differential DNA binding activity, cofactor recruitment, and transcriptional outcomes but we view both possibilities as likely within the emerging principals of allostery. Mutational experiments with so many possible interacting modes might prove challenging but would be best guided by in-silico mutation to examine which amino acids among the hot spots / interface amino acids provided are the best candidates.

### Allostery in steroid hormone receptors

DNA allosterically influences GR whereby the exact GRE sequence including the spacer allows GR to bind in slightly different conformations repositioning key DBD interfaces [75, 76]. Consequently, information is transmitted bidirectionally through the D-loop and lever arm to additional domains allowing the entire protein to operate as an integrated unit [37, 39, 76, 77]. A consequence of this plasticity is that different DNA binding sites display differential receptor domain dependency [37, 78], and therefore require differential chromatin remodelling proteins [79] and coregulators [76, 80] for receptor activity. Individual binding sites may differentially re-orientate protein-protein contacts exploiting the variety of interfaces we predict to be available for MR and GR to interact, exposing context-dependent surfaces for cofactor recruitment. Allostery through homo- and hetero-oligomerisation is common to other receptor systems, a specific ligand for one member of the G-protein coupled chemokine receptor family can influence the function of another receptor type binding an alternative ligand through allosteric transfer of information [62]. It would be unsurprising should it transpire that evolution has applied these mechanisms to additionally confer signal diversity in glucocorticoid signalling.

### Higher order MR-GR complexes

Classically GR and MR dimerize at or before nuclear entry and load at DNA as homodimers or heterodimers to control gene regulation. Microscopy has recently confirmed early glycerol density gradient centrifugation studies [16] showing ligand activated GR is an intranuclear dimer for several cell types including 3617 [58, 81]. Having demonstrated using proximity ligation assay that the interaction of MR-GR in 3617 cells did not require an intact D-loop interface expected to mediate this interaction [12, 25], we show that molecular brightness (ε) for EGFP-GR was slightly increased by mCherry-MR co-expression (Figs 6 and 7). The molecular stoichiometry of ligand activated MR has generally been assumed to be a dimer [25, 82], but whilst appearance of intermolecular BRET following aldosterone treatment is consistent with at least a higher order than a monomer [83], a dimer has never been confirmed. The intranuclear oligomerisation state of mCherry-MR was not altered by co-expression with EGFP-GR. Together these results support MR-GR interaction the nucleoplasm but not as a 1:1 heterodimer mediated through the D-loop.

Although with one exception [33], MR-GR interactions have currently only been described at DNA. However, the majority of nuclear receptors are not DNA-bound [84] so the relationship between the molecular brightness in the nucleoplasm and at the MMTV array was examined. Anticipating heterodimer formation between chromatin-associated, interacting MR-GR would be associated with a lower ε at the array relative to the nucleoplasm (where brightness increased or stayed the same), we instead observed higher oligomerisation states for both MR and GR at the array. Interestingly, GR tetramerization at the array was recently reported [61]. Our current data imply that far from heterodimer formation, MR/GR may also assume a higher order complex at DNA, possibly supported through the variety of interfaces predicted. Complexes of higher order than dimers for RXR, PR and AR have also been reported so may not be unique to MR-GR [61, 85]. Although N&B and PLA data were not consistent with a sizable population of the interacting MR/GR molecules behaving as 1:1 MR-GR heterodimers in the nucleus or at the chromatinised MMTV array, these techniques report the average population state and so we cannot rule out a small population of true heterodimers accessing binding sites elsewhere in the nucleus below detection limits. Alternative complexes proposed by our structural predictions appear likely, while a variety of conformations additionally release MR-GR DNA binding activity from conventional palindromic GREs and may explain how these complexes access direct halfsite repeats spaced by six [29] or nine bases [30].

### Transcriptional implications

Alternative MR-GR interactions that adjust cofactor requirements via the allosteric production of diverse regulatory surfaces may explain the variety of outcomes observed at the transcriptional level for these complexes. Inhibition by MR of the GR-mediated response is commonly observed [25, 28, 31, 86], but synergistic or augmented actions of MR-GR interaction relative to GR alone have also been reported [26, 29, 64]. Potentially consistent with multiple binding modes, findings have not been consistent within the same cells, MR-GR inhibits relative to GR alone at multimers of the GRE palindrome, but not at single GREs [25]. Only one study has examined MR-GR cooperativity in the regulation of endogenous genes, reporting augmented GR-mediated responses [64]. The functional role of MR-GR interactions is far from settled.

In summary we show MR-GR interactions are attainable at DNA and can be achieved without the classical D-loop interface. Structurally supporting a variety of alternative complexes we propose structural malleability or allostery as a feature of MR-GR cooperative function contributing to functional diversity in glucocorticoid-mediated gene regulation. The interacting MR-GR arrangement may depend on the availability of a broad spectrum of ligands, the DNA binding site, and non-receptor interacting partners. Drug discovery has targeted higher affinity, longer acting, GR-selective compounds for anti-inflammatory activity and steroid replacement therapy. If MR and GR interact in co-expressing cell types to mediate at least some actions of endogenous glucocorticoid hormones present approaches are likely to be compromised by a significant risk of side effects. Indeed, reduced MR activity is a feature of such synthetic compounds [87] and has been associated with neuropsychiatric disorders [88]. With the balance of MR/GR activities being important for normal function of the brain [46], heart [89] and adipose tissue [90], and imbalanced activities associated with vulnerability to disease states, the behaviour of interacting MR-GR as a functional transcriptional complex may add new dimensions to balanced corticosteroid receptor signalling in such tissue types.

## Materials and methods

### Sample preparation

#### Plasmids and hormones

Full length rat MR was obtained by PCR amplification of plasmid 6RMR [14] (kind gift from Prof. David Pearce, University of California, San Francisco). The PCR product was inserted into the BspEI and SacII sites of pEGFP-C1 or pmCherry-C1 (Clontech, Mountain View, CA) to obtain pEGFP-C1-rMR or pmCherry-C1-rMR. In each case the fluorophore was tagged to the N-terminal of the MR. The linker in the EGFP variant was SGLRS and YKSGLRS in pmCherry-C1-rMR. pEGFP-C1-rGR_C656G_ and pmCherry-C1-rGR_C656G_ were produced by sub-cloning rat GR with a C656G mutation into pEGFP-C1 (linker SGLRSRGAGAGAGAGAISALI) or pmCherry-C1 (linker LYKSGLRSRGAGAGAGAGA). pEGFP-rGRwt was produced from pEGFP-C1-rGR_C656G_ by site directed mutagenesis according to the manufacturer’s instructions, correcting the C656G site using a QuikChange XL kit (Agilent, Santa Clara, CA). pEGFP-AR-mCherry (gift from Ty Voss, LRBGE, NCI) contained an androgen receptor tagged at the N-terminal with EGFP and at the C-terminal with mCherry. The A207K variant of EGFP and corresponding EGFP-rGRwt plasmid were produced by site directed mutagenesis. Untagged pC1-rMR was produced by gel purification of the mCherry-containing AgeI/BspEI fragment of pmCherry-C1-rMR away from the rest of the vector and ligation of compatible sticky ends. The Kozak sequence was then restored by site directed mutagenesis. The equivalent pC1-rGRwt was produced by PCR amplification of GRwt from pEGFP-rGRwt and insertion into the NdeI/ApaI cut sites of the same parent plasmid. Combined D-loop salt bridge and hydrogen bonding defective mutations of GR and MR were produced using site direct mutagenesis of pC1-rGRwt and pC1-rMR. pFC31Luc contains the mouse mammary tumor virus long terminal repeat (MMTV LTR) driving firefly luciferase expression and was a gift from Helene Richard-Foy and Gordon Hager [91]. pRL-CMV (Promega, Southampton, UK) contains a CMV promoter driving Renilla luciferase as a transfection control. pcDNA3 (Invitrogen, Waltham, MA) provided an empty vector with a CMV promoter. Several plasmids described were constructed at the Laboratory of Receptor Biology and Gene Expression, CCR, NCI, MD and obtained in the UK under material transfer agreement (gift from Gordon Hager). Corticosterone, dexamethasone, prednisolone, aldosterone, spironolactone, mifepristone and testosterone (Sigma-Aldrich, St Louis, MO; or Gillingham, UK) were dissolved in ethanol and diluted for the final concentrations indicated so that final ethanol concentrations were 0.01% consistent with vehicle controls.

#### Cell lines and culture

3617 cells have been described previously [48] to contain a tandem head-to-tail array of 200 copies of the MMTV LTR driving a viral Harvey Ras cDNA, and a GFP-tagged rat GR with the C656G mutation. Derivative 3617ChMR was produced by retroviral transduction of 3617 and 14 days selection in hygromycin (Life Technologies, Carlsbad, CA). Briefly, the mCherry-rMR between AgeI/MfeI of pmCherry-C1-rMR was sub-cloned into pRevTRElink [92] and the resulting plasmid, pRevTRElink-mCherry-rMR, transfected into Phoenix packaging cells [93]. Infection of 3617 with resulting retrovirus allowed integration of a tetracycline-sensitive mCherry-rMR. Both parent and derivative cell lines were transferred to the UK under material transfer agreement from Gordon Hager, NCI. The 3617_M20-_ derivative was produced by CRISPR/Cas9-mediated homologous recombination to remove the epitope for the antibody anti-GR M-20. Briefly, complementary DNA oligos were annealed and inserted in to the BbsI site of plasmid pX330 (Addgene #42230) containing Cas9 by standard protocol [94]. The resulting construct was co-transfected (1:1) with a pcDNA3 plasmid containing a donor segment placing the blasticidin resistance gene *Bsd* in frame with the GR start codon. Two homology arms ensured the correct placing and transfected cells were selected with blasticidin (InvivoGen, San Diego, CA) before monoclone isolation.

3617, 3617ChMR and COS-1 cells were maintained in DMEM high glucose (Life Technologies, CA; Thermo Fisher, Paisley, UK) supplemented with 10% FBS (Atlanta Biologicals, Flowery Branch, GA; Thermo Fisher, UK) and 1% L-glutamine (Life Technologies, CA; Thermo Fisher, UK). Expression of the integrated GFP-GR (and mCherry-MR for 3617ChMR) was prevented during general culture by growth in 5 μg/ml tetracycline (Sigma). Neuro-2a cells (N2a, ECACC, #89121404, Public Health England) were maintained in DMEM high glucose supplemented with 7.5% FBS, 1% L-Glut, 0.4 mM L-aspartic acid, 0.4 mM L-glutamic acid, 0.3 mM cysteine, HCl, 0.4 mM L-alanine, 0.45 mM asparagine, and 0.4 mM L-proline as previously described [95].

Seeding of cells for experiments was performed in growth media wherein the FBS component was replaced with charcoal stripped serum (GE Healthcare Life Sciences, Logan, UT; Thermo Fisher, UK). For 3617 and derivatives 5 μg/ml tetracycline was either included to prevent expression of integrated fluorescent receptors or omitted to allow expression.

#### Animals and treatments

Male Sprague Dawley rats (200-250g) were group housed (3–4 per cage) with food and water *ad libidum*. Randomly allocated groups were given a restraint stress for 30 min in Perspex restraining tubes with screw-fix ends, or intraperitoneal injection of corticosterone-HBC (3 mg/kg corticosterone equivalent) or HBC vehicle in 0.9% saline (both Sigma-Aldrich, UK) 30 min before deep isoflurane anaesthesia and decapitation. Some animals underwent bilateral adrenalectomy via the dorsal approach under balanced isoflurane anaesthesia (Merial Animal Health Ltd, Woking, UK) to remove endogenous corticosterone from the circulation. Adrenalectomised animals received subcutaneous dexamethasone and carprofen (Rimadyl, Pfizer, Walton Oaks, UK) for pain relief and were recovered for 5 days post-surgery on 15 μg/ml corticosterone in 0.9% saline drinking solution to maintain isotonic levels. Drinking solution was replaced 12 h prior to injection experiments with 0.9% saline. Brains were removed post-mortem and the hippocampus micro-dissected on ice, then flash frozen in liquid nitrogen. All animal procedures were performed in accordance with the revised UK Animals (Scientific Procedures) Act of 1986 and EU Directive 2010/63/EU following approval of the University of Bristol Animal Welfare and Ethics Review Body under project license number 30/3114 granted by the UK Home Office.

### Molecular biology

#### Co-immunoprecipitation

Cells were washed once in ice cold 1X PBS then scraped into 1ml 1X PBS supplemented with 50X cOmplete protease inhibitor cocktail (no EDTA, Roche Diagnostics, Indianapolis, IN; or Burgess Hill, UK), and phosphatase inhibitor 2mM sodium fluoride (NaF, Sigma). Co-immunoprecipitation was performed according to the method of Nishi [32]. Cells were pelleted by centrifugation (2400×g, 5 min, 4°C) and lysed in 1ml coIP buffer [50mM Tris-HCl pH 7.5, 500mM NaCl, 2mM EDTA, 1% IGEPAL-CA630, 0.5% Triton X-100] supplemented with 50X cOmplete protease inhibitor cocktail (no EDTA) and NaF. Efficient lysis was ensured by passing material 10 times through a 25-gauge needle and incubation for 5 min on ice.

Dissected hippocampus flash frozen in liquid nitrogen was thawed 5 min on ice in 1ml coIP buffer (supplemented as above plus 0.2mM phosphatase inhibitor sodium orthovanadate (NaVan, Sigma)). Brain material was homogenized on ice using a Polytron PT-1200-E with attachment PT-DA 05/2EC-E085 (Kinematica AG, Lausanne, Switzerland). Full lysis was ensured by 20 min incubation of homogenised material on ice.

Cell line or tissue samples were cleared of debris by centrifugation (17,000×g, 4°C, 10 min) and supernatant transferred to a fresh tube before preclearing with 60μl protein A agarose beads (Merck Millipore, Billerica, MA; or Watford, UK) for 1 hr at 4°C under constant rotation. Supernatant was transferred to a fresh tube before addition of 2μg (cells) or 10μg (tissue) immunoprecipitation antibody. Antibodies were non-immune rabbit IgG (sc-2027 X) or rabbit anti-GR M-20 polyclonal (sc-1004 X) (both Santa Cruz Biotechnology, Dallas, TX). Following 1 hr rotation at 4°C protein complexes were collected onto 60μl protein A agarose beads for an additional 1 hr, washed 3 times (cells) or 5 times (tissue) in coIP buffer (supplemented as above, 5 min washes), then eluted by shaking for 20 min, room temperature (RT) into 20μl 1X NuPAGE LDS Sample Buffer (Thermo Fisher, Waltham, MA; or Paisley, UK) supplemented with 0.1M DTT (Sigma).

Eluted proteins were denatured at 98°C for 5 min, separated from beads by centrifugation (3500×g, 2 min, RT), then loaded run on NuPAGE Novex 3–8% Tris-Acetate protein mini gels (Thermo Fisher). Proteins were transferred onto PVDF membrane (Thermo Fisher) in tris-glycine transfer buffer (Cell Signaling, Danvers, MA; or Leiden, Netherlands). Non-specific sites were blocked with 5% non-fat milk in 1X TBST, then probed overnight at 4°C using mouse anti-MR monoclonal antibody 1D5 (1:4000, gift from Professor Celso Gomez-Sanchez, University of Mississippi Medical School). Membranes were washed 3× in TBST, probed with sheep anti-mouse secondary conjugated to HRP (1:4000, GE Healthcare, Little Chalfont, UK, 2 hrs, RT), then washed a further 3× before immunoreactive bands were visualized with SuperSignal West Pico Chemiluminescent Substrate (Thermo Fisher).

#### Proximity ligation assay (PLA)

3617 or 3617_M20-_ were seeded onto 35 mm Nunc glass bottom dishes (Thermo Fisher, UK). Transfection (as above) with pC1-rGRwt plus either empty vector pcDNA3, or pC1-rMR at a ratio of 1:1 provided a negative control condition expressing only GR, or an MR+GR co-expression test condition. For some experiments, plasmids containing mutations in the D-loop of either receptor were used instead of wildtype proteins. Media was changed after 4 hrs, then 24 hrs following transfection, 100nM corticosterone was added for 45 min before cells were fixed in 4% paraformaldehyde for 15 mins on an orbital shaker. Cells were washed 3 times in 1X PBS before blocking with Duolink Blocking Solution (Duolink In Situ Red Mouse/Rabbit Kit, Sigma, Gillingham, UK), applied to the imaging area for 2 hrs at 37°C in a humidified chamber. Blocking was followed by 2×5 min washes with 1X PBS prior to incubation with primary antibodies anti-GR M-20 and anti-MR 1D5 (each 1:500) for 72 hrs at 4°C in 1X PBS with 0.3% Triton-X100 to aid permeabilization.

Cells were washed 3×10 min in 1X PBS to remove unbound primary antibody prior to PLA. All incubations were performed in a humidified chamber at 37°C and according to the Duolink In Situ Detection Reagents Red kit instructions (#DUO92008, Sigma-Aldrich, Gillingham, UK). Briefly, blocked cell preparations were incubated with PLA probes corresponding to the primary antibodies (Duolink In Situ PLA Probe Anti-Mouse MINUS #DUO92004, and Duolink In Situ PLA Probe Anti-Rabbit PLUS #DUO92002, Sigma) for 60 minutes, washed 4×5 min in 1X Wash Buffer A before incubation with DNA ligase diluted in ligation buffer for 30 min. Further washing (4×2 min in 1X Wash Buffer A) preceded incubation with a DNA polymerase diluted in Amplification Buffer for 100 min. Samples were washed 4×10 minutes in 1X Wash Buffer B during which dishes were covered with foil to prevent fluorophore bleaching. During the first wash DAPI (4',6'-diamidino-2-phénylindole) was added at 1μg/ml to stain nuclei (Sigma, Gillingham, UK). A final wash (1 min, 0.1X Wash Buffer B) removed excess salt and detergent and preparations were stored in 1X PBS at 4°C for imaging.

Fluorescence was visualized using a Lecia SP5-II AOBS laser scanning microscope attached to a Lecia DM I6000 inverted epifluorescence microscope fitted with a 40X PL APO CS oil objective, NA 1.3 (Wolfson Bioimaging Facility, University of Bristol). Excitation was with a 50 mW 405 nm diode laser (DAPI) and a 20 mW solid state yellow laser (561 nm, Duolink Red). GaAsP detectors in photon counting mode captured DAPI (410–460 nm) and Duolink Red (570–650 nm) signals.

#### Fluorescent immunohistochemistry

Cell plating, transfection, treatment and fixation was performed as above. Blocking of non-specific binding was performed in 0.1 M PBS, pH 7.4 containing 0.3% Triton X-100 and 2% bovine serum albumin, fraction V (Sigma) for 2 hrs at RT. Washes (2×5 min, 1X PBS) removed blocking solution before incubation with primary antibodies and further washing as described above. Secondary antibodies donkey anti-rabbit Alexa Fluor-488 (#ab150061) and goat anti-mouse Alexa Fluor-594 (#ab150120) were incubated with cell preparations each at 1:1000 for 2 hrs at RT in 0.1 M PBS, pH 7.4 containing 0.3% Triton X-100 and 2% bovine serum albumin, fraction V (Sigma). Washes (3x10 min in 1X PBS) removed un-bound secondary antibodies before imaging on a Lecia SP5-II AOBS laser scanning microscope.

#### Chromatin immunoprecipitation (ChIP)

3617 cells were seeded at 6 million cells per 15 cm dish in media containing charcoal stripped serum without tetracycline allowing integrated GFP-GR_C656G_ to express. After overnight incubation and reattachment, cells were transfected with 10μg pmCherry-C1-rMR (jetPRIME, Polyplus) and media changed after 4 hrs. After 24 hrs to express both integrated GFP-GR_C656G_ and transfected mCherry-rMR, cells were subjected to a pulse of 100nM corticosterone [4]. Washout media changes were performed as above by means of a modified incubator door (University of Bristol, Joint Faculty Workshop) to minimize cellular stress.

At the time points indicated during the corticosterone pulse, cells were fixed directly in plates for 10 mins with gentle rocking by addition of 18.5% formaldehyde to 1% final concentration. Addition of glycine to 0.125M for 5 mins quenched fixation, then cells were washed 3 times with ice-cold PBS before lysis in 2% SDS lysis buffer [2% SDS, 10 mM EDTA, 50 mM Tris-HCl, pH 8.1] supplemented with cOmplete protease inhibitors (Roche), 2 mM NaF, and 0.2 mM NaVan. Lysates were passed through a 25-gauge needle 5 times before sonication (4×10 second pulses, Branson Sonifier 450, 10% output, Branson Ultrasonics, Danbury, CA), then cleared of cellular debris by centrifugation (10 min, 11,600×g, 4°C). 1% of chromatin inputs were collected and stored for downstream processing.

70μg chromatin in 100μl lysis buffer was diluted 1:10 in ChIP dilution buffer supplemented with cOmplete protease inhibitors, 2 mM NaF, and 0.2 mM NaVan, and pre-cleared with 25μl binding control agarose beads (bab-20, Chromotek, Munich, Germany). Overnight immunoprecipitation of the GFP and mCherry tags was performed with 25μl GFP-Trap^®^_A (gta-20, Chromotek) or 25μl RFP-Trap^®^_A (rta-20, Chromotek). Control beads with no antibody attachment were used as a negative control. Beads were washed with low salt wash buffer [20mM Tris-HCl pH 8.1, 150mM NaCl, 2mM EDTA, 1% Triton X-100, 0.1% SDS], twice with high salt wash buffer [20mM Tris-HCl pH 8.1, 300mM NaCl, 2mM EDTA, 1% Triton X-100, 0.1% SDS], once with LiCl wash buffer [10mM Tris-HCl pH 8.1, 0.25M LiCl, 1mM EDTA, 1% deoxycholic acid, 1% IGEPAL-CA630] and twice with TE buffer [10mM Tris-HCl pH 8.0, 1mM EDTA]. Protein/DNA complexes were recovered by shaking in elution buffer [1% SDS, 0.1M NaHCO_3_] before samples and inputs had cross-links reversed (adjusted to 0.2M NaCl, 65°C, overnight). Contaminants were removed by digestion with DNase-free RNase (Roche), and proteinase K (Thermo Fisher) prior to phenol-chloroform extraction and ethanol precipitation to recover DNA fragments into nuclease free water.

Quantitative PCR of GRE-containing promoter regions in ChIP samples was performed on a StepOnePlus Real-Time PCR System using Fast SYBR green master mix (Applied Biosystems Inc, Foster City, CA). Primers were; forward 5'-TTAAGTAAGTTTTTGGTTACAAACT-3' and reverse 5'-TCAGAGCTCAGATCAGAACCTTTGATACC-3' for *MMTV nucleosome B*, forward 5’-TCTAACTCGCCACCTCCTCA-3’ and reverse 5’-CCAACTAATCTCCGAGAACA-3’ for *Sgk1*, and forward 5’-GGGACCCCCTTCCTCCTAAC-3’ and reverse 5’-AGCGCACTAGGGAACATCGT-3’ for *Per1*.

#### Luciferase assay

Performed using the Dual-luciferase reporter assay system according to the manufacturer’s instructions (Promega, Southampton, UK). COS-1 cells were seeded in charcoal stripped serum containing media and transfected with the plasmids indicated along with pRL-CMV and pFC31Luc to provide transfection controls and a GR-activatable luciferase reporter. Lysates were collected for analysis 24 hrs later.

### Imaging methodology

#### Number and brightness assay and cross-correlation N&B

3617 cells were seeded to NUNC Lab-Tek chamber slides (Thermo Fisher, CA) or 35 mm CELLview^™^ dishes with glass bottoms (Greiner Bio-One, Stonehouse, UK) in media containing tetracycline to prevent expression of integrated fluorescent receptors. N2a cells were seeded in the same manner without tetracycline but dishes were pre-coated with poly-L-lysine to aid adherence. The next day cells were transfected with plasmids coding fluorescent proteins using jetPRIME DNA/siRNA transfection reagent according to the manufacturer’s instructions (Polyplus-transfection, New York, NY; Illkirch, France). Given the mismatch in quantum yield EGFP-GR_C656G_ and mCherry-MR were transfected at 1:2 ratio. Media was changed 4 hrs later to aid cell recovery, and experiments were conducted 24 hrs following transfection. Corticosterone was applied for a minimum of 30 min before imaging.

For cross-correlation number and brightness assay (USA) 256 pixel × 256 pixel frames were scanned on a Zeiss LSM780 laser scanning microscope with an environmental chamber and a 63X oil immersion objective, NA 1.4 (Carl Zeiss Inc, Thornwood, NY). For each cell examined a stack of 150 raster scan images was collected with frame intervals of 0 s, pixel size 80nm and pixel dwell time 6.3μs. The frame time was 0.97 s guaranteeing independent sampling of fluorescent molecules [61]. An argon laser tuned to 488nm and HeNe 594nm laser provided excitation for EGFP and mCherry respectively while emissions were collected in green and red channels simultaneously using gallium arsenide phosphide (GaAsP) detectors in photon counting mode. EGFP emissions were collected in the range 491-571nm and mCherry in the range 616-696nm. When performed in the UK, imaging was performed on a Leica SP8 AOBS confocal laser scanning microscope attached to a DM I6000 inverted epifluorescence microscope, fitted with a 100X oil immersion objective, NA 1.44 (Leica Microsystems, Milton Keynes, UK). The chamber was temperature controlled, maintained at 37°C, and CO_2_ was delivered into the imaging chamber at a constant rate, but setting precisely to 5% was not achievable. Imaging settings were as described above though the frame time was longer (1.677 s). A 65 mW 488nm argon laser and 2 mW HeNe 594nm laser provided excitation lines, with emissions collected simultaneously using GaAsP detectors in photon counting mode.

For analysis 16-bit images were exported and loaded into the N&B routine within SimFCS software developed by the Laboratory for Fluorescence Dynamics (University of California, Irvine, CA). The first 10 frames from each series were discarded to reduce overall bleaching artefacts and the pixels representing different regions of the cell were selected by thresholding before determining the Bcc or ε values as described elsewhere [52].

#### MMTV array imaging during pulsatile stimulation of 3617ChMR

3617ChMR cells were seeded onto coverslips without tetracycline to allow integrated fluorescent MR and GR to express for 27 hrs prior to treatment. Corticosterone was applied at the doses indicated in 0.01% final ethanol for 20 min before washout by media change under 95% humidity, 37°C and 5% CO_2_ to mimic ultradian pulses [4]. Cells were fixed at the time points indicated in 4% paraformaldehyde within a 1:1 mixture of media containing charcoal stripped serum and 0.1M DPBS, pH 7.4 (Gibco). Cells were washed in 0.1M DPBS and coverslips mounted to sides using VectaShield antifade mounting medium with DAPI (Vector Laboratories Inc, Burlingame, CA).

Imaging utilised a DeltaVision System attached to an inverted Olympus IX70 fluorescence microscope fitted with a 60X, NA 1.4 oil objective (Laboratory of Receptor Biology and Gene Expression Core, National Cancer Institute, MD). An axillary magnification of 1.5 was used to collect image stacks in the z direction with a step size 0.3μm. Focal planes covered the entire nucleus and images were captured with a Cool Snap HQ camera (Photometrics, Roper Scientific, Tucson, AZ). Automated filter wheels and a polychroic light splitter allowed capture of each fluorophore. Constrained iterative image deconvolution was performed to reassign out of focus light with Softworx Explorer Suite (Applied Precision, Issaquah, WA).

Images of at least 130 complete cells were collected for each time point within 7–15 fields of view. Cells with arrays marked by fluorescent MR and GR were assessed by human observer and calculated as a percentage of the total number of observable cells in the fields of view. Each independent experiment contained 3 repeats of the time points shown.

#### Fluorescence recovery after photobleaching (FRAP)

3617 cells grown in tetracycline to prevent integrated receptor expression were transiently transfected with pEGFP-C1-rMR. Allowing 24 hrs for protein expression FRAP was performed as previously described [4], photobleaching the fluorescent MR protein over the array site, then following fluorescent recovery time of the same location.

### PRISM computational modelling of protein-protein interactions

The PRISM: Protein Interactions by Structural Matching tool [59, 60] was used to model MR-GR interactions, on an Ubuntu 14.04.6 LTS (GNU/Linux 3.13.0-170-generic x86 64) operating system. The PRISM algorithm is a template-based protein interaction prediction tool that utilises interaction structures available in the Protein Data Bank [96] as templates to predict structural interaction between two query proteins. PRISM generates multiple possible models using template interfaces that show a good match to any possible interaction interface between the two query proteins. Rigid docking results are then flexibly refined to resolve steric clashes, outputting a pdb file of the predicted interaction structure and a list of interacting amino acids in the predicted interface. Publications of the PRISM tool have been cited by 287 papers, including recent independent publications from 2017 and 2019 that have validated PRISM predictions via experimental methods [97, 98].

Binding energies of PRISM predictions were calculated using ZRANK [99], an optimised energy function providing an arbitrary energy score for comparative analyses allowing ranking of multiple protein docking predictions. Energies were computed for all PRISM interaction predictions utilising interfaces from the nuclear receptor protein family. For each possible interface per query pair of proteins, the prediction with the lowest energy score for that interface was used. For cross-checking and selection of the most probable models, the PDBePISA server of the European Bioinformatics Institute was used [100]. PDBePISA provided calculations for the interface area in Å^2^, the solvation free energy Δ^i^G in kcal/mol and the Δ^i^G P-value. According to PDBePISA, P>0.5 means interface is less hydrophobic than it could be, and likely to be an artefact of crystal packing. P<0.5 indicates significant hydrophobicity, implying that the interface surface can be interaction-specific. All the PRISM predictions provided have been ensured to have P values below 0.5, meeting this significance threshold.

DNA binding and ligand binding domain structures of GR and MR were obtained from the Protein Data Bank. Rat homology models were constructed using Swiss Model [101] where only the human or mouse structures were available. To ensure that homology modelling of structures would be reliable, a high sequence identity between rat and human or mouse homologs was confirmed using protein BLAST [102].

Hot spot amino acid residues that contribute most of the binding energy to the protein-protein interaction interfaces [103, 104] predicted by PRISM were computed using HotRegion [105]. HotRegion additionally identifies the regions of hot spot clusters important for the stability of protein complexes.

To determine mutations with deleterious effects on the interaction between the DNA binding domains of GR and MR, changes in binding affinity (ΔΔG) were calculated using several tools; FoldX [106, 107], BeatMusic [108], mCSM [109] and MutaBind [110].

### Statistical analysis

Statistical procedures are described in the text or figure legends.

## Supporting information

S1 Fig MR DNA occupancy is prolonged during corticosterone washout but the receptor displays rapid ‘hit and run’ dynamics. (A) GFP-GR_C656G_ and mCherry-MR do not respond to ethanol vehicle (0.01% final concentration, 30 min). (B) 3617 cells +tetracycline transiently transfected with EGFP-tagged rMR and treated with 100 nM corticosterone. MMTV array bound EGFP-MR was photobleached with a high intensity laser. Stable MR DNA binding produces a permanent dark spot over the array. Conversely, fluorescent recovery of EGFP-rMR was rapid indicating MR turnover at a chromatinised DNA followed a ‘hit and run’ mode of receptor action. Mean ± SEM, N = 14. (C) Simulated 20 min pulse of 5 nM corticosterone (physiological ultradian pulse range) in 3617ChMR cells without tetracycline. Four complete media changes 2 min apart ensured residual hormone levels were as low as possible. MMTV array loading of GFP-GR_C656G_ occurred only at the pulse peak (levels only just measurable at this dose). Loading of mCherry-MR was evident at the pulse peak and a majority remained DNA-bound at 60 min consistent with previous experiments. Loss of mCherry-MR from DNA occurred slowly and was largely complete between 120 and 180 min after pulse initiation, transcending the inter-pulse interval. One experiment of N = 3, Mean ± SEM.
(TIF)

S2 Fig PLA antibody specificity controls. (A) 3617 cells do not express MR but contain endogenous mouse GR. To avoid interference from endogenous GR CRISPR-Cas9 was used to remove the antibody recognition epitope from the first exon of the GR. A guide RNA positions Cas9 close to the start codon of the mouse GR which runs in the antisense direction on chromosome 18, and CRISPR-mediated DNA editing was achieved by homologous recombination between two homology arms one in the GR promoter region and the other positioned toward the end of the GR poly-Q repeat, removing amino acids 3–90 from the protein coding sequence in which the anti-GR antibody epitope lies. The initiating methionine and following aspartic acid were preserved. Deleted sequence was replaced with the blasticidin resistance gene *Bsd* in frame with the endogenous GR start codon allowing isolation of a monoclonal cell population. (B) Western blot showing the loss of anti-GR M-20 detection of the GR in 3617_M20-_ cells compared to the parental cell line. (C) 3617_M20-_ cells were a negative baseline for immunohistochemistry using the anti-GR M-20 antibody. Cells were transfected +tetracycline with full length rat MR or GR or pcDNA3, corticosterone treated (100 nM, 45 min) and fixed for immunohistochemistry. Primary antibodies were applied as described, all samples received both Alexa Fluor-labelled secondary detection antibodies. MR and GR detection with the primary antibody pair used for PLA was clear and specific demonstrating no cross-reactivity. Scale bar = 100 μm.
(TIF)

S3 Fig Representative images for ccN&B experiments in which alternative endogenous and synthetic ligands for MR and GR were applied to transfected 3617 cells +tetracycline. (A) Application of 100 nM of the compounds indicated and compared to corticosterone. (B) Application of combinations of agonists and antagonists. Dexamethasone (Dex) 10 nM + aldosterone (Aldo) 10 nM, spironolactone + RU486 (1 μM each), aldosterone + RU486 and corticosterone + RU486 (10 nM MR-targeted agonist, 1 μM GR-targeted antagonist) were compared to 100 nM corticosterone. Treatments for minimum of 30 min before imaging. Scale bars = 5 μm.
(TIF)

S1 Table Interacting residues and hot spots for the predicted classical heterodimer interface in receptor DBDs (Fig 5A). Interacting residues on GR are on the left and those on MR on the right. Hot spot residues are highlighted in yellow. Both MR and GR D-loop residues make contacts with residues within and outside the D-loop of the opposing receptor. Aside from the cysteine residues that coordinate the overall conformation of the second zinc finger, Ala-477 on GR and Ala-639 on MR are considered hot spot residues with the highest pair potentials and therefore the single residues with the highest probability of disrupting the interface if mutated.
(XLSX)

S2 Table Effect of individual amino acid mutations alone or in combination on the classical D-loop interface between MR-GR. Predictions are for GR changes and show the average ΔΔG score from alternative mutation analysis software. Colour coding reflects the severity of the change in interaction potential with the darkest blue the strongest predicted change. Note that A477T is the GRdim mutation first demonstrated as a natural mutation in human AR.
(DOCX)

S3 Table Interacting residues and hot spots for alternative predicted MR-GR interaction modes of the DBDs shown in Fig 8A and 8B. Each sheet references the figure number and part in which the model is presented, then the interface name.
(XLSX)

S4 Table Energy and area values of the interface templates matched from the PDB. (XLSX)

S5 Table Interacting residues and hot spots for alternative predicted MR-GR interaction modes of the LBDs shown in Fig 8C. Each sheet references an alternative interface predicted by PRISM for the MR and GR LBDs.
(XLSX)

S1 Raw Images Uncropped source images for western blots presented. (PDF)
