# Supplementary figures and images for: Beyond the heterodimer model for mineralocorticoid and glucocorticoid receptor interactions in nuclei and at DNA
Source: PLoS One. 2020 Jan 10;15(1):e0227520. doi: 10.1371/journal.pone.0227520 (PMC6953809; doi:10.1371/journal.pone.0227520)

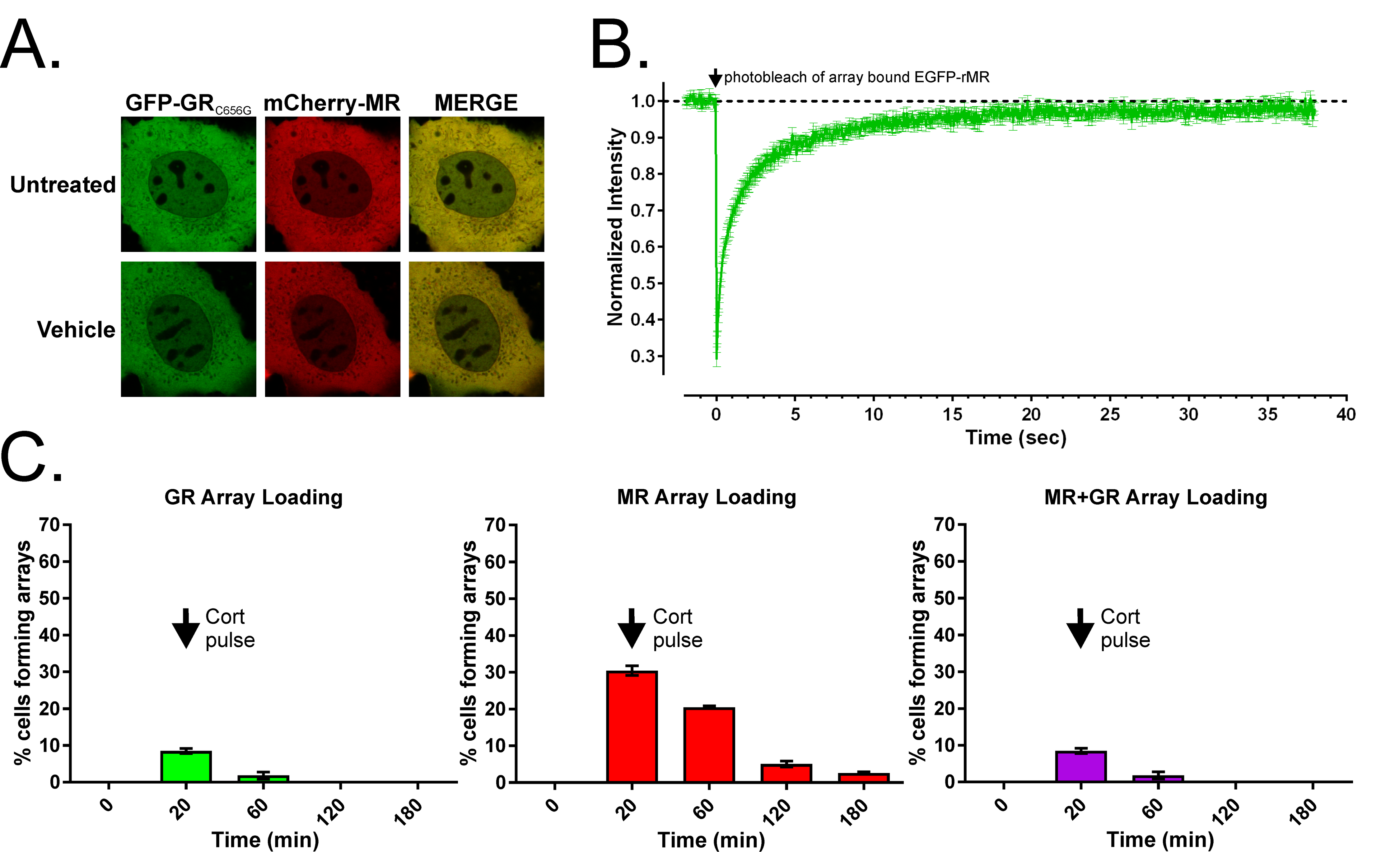

Supplement: S1 Fig — (A) GFP-GRC656G and mCherry-MR do not respond to ethanol vehicle (0.01% final concentration, 30 min). (B) 3617 cells +tetracycline transiently transfected with EGFP-tagged rMR and treated with 100 nM corticosterone. MMTV array bound EGFP-MR was photobleached with a high intensity laser. Stable MR DNA binding produces a permanent dark spot over the array. Conversely, fluorescent recovery of EGFP-rMR was rapid indicating MR turnover at a chromatinised DNA followed a ‘hit and run’ mode of receptor action. Mean ± SEM, N = 14. (C) Simulated 20 min pulse of 5 nM corticosterone (physiological ultradian pulse range) in 3617ChMR cells without tetracycline. Four complete media changes 2 min apart ensured residual hormone levels were as low as possible. MMTV array loading of GFP-GRC656G occurred only at the pulse peak (levels only just measurable at this dose). Loading of mCherry-MR was evident at the pulse peak and a majority remained DNA-bound at 60 min consistent with previous experiments. Loss of mCherry-MR from DNA occurred slowly and was largely complete between 120 and 180 min after pulse initiation, transcending the inter-pulse interval. One experiment of N = 3, Mean ± SEM. (TIF) [file pone.0227520.s001.tif]

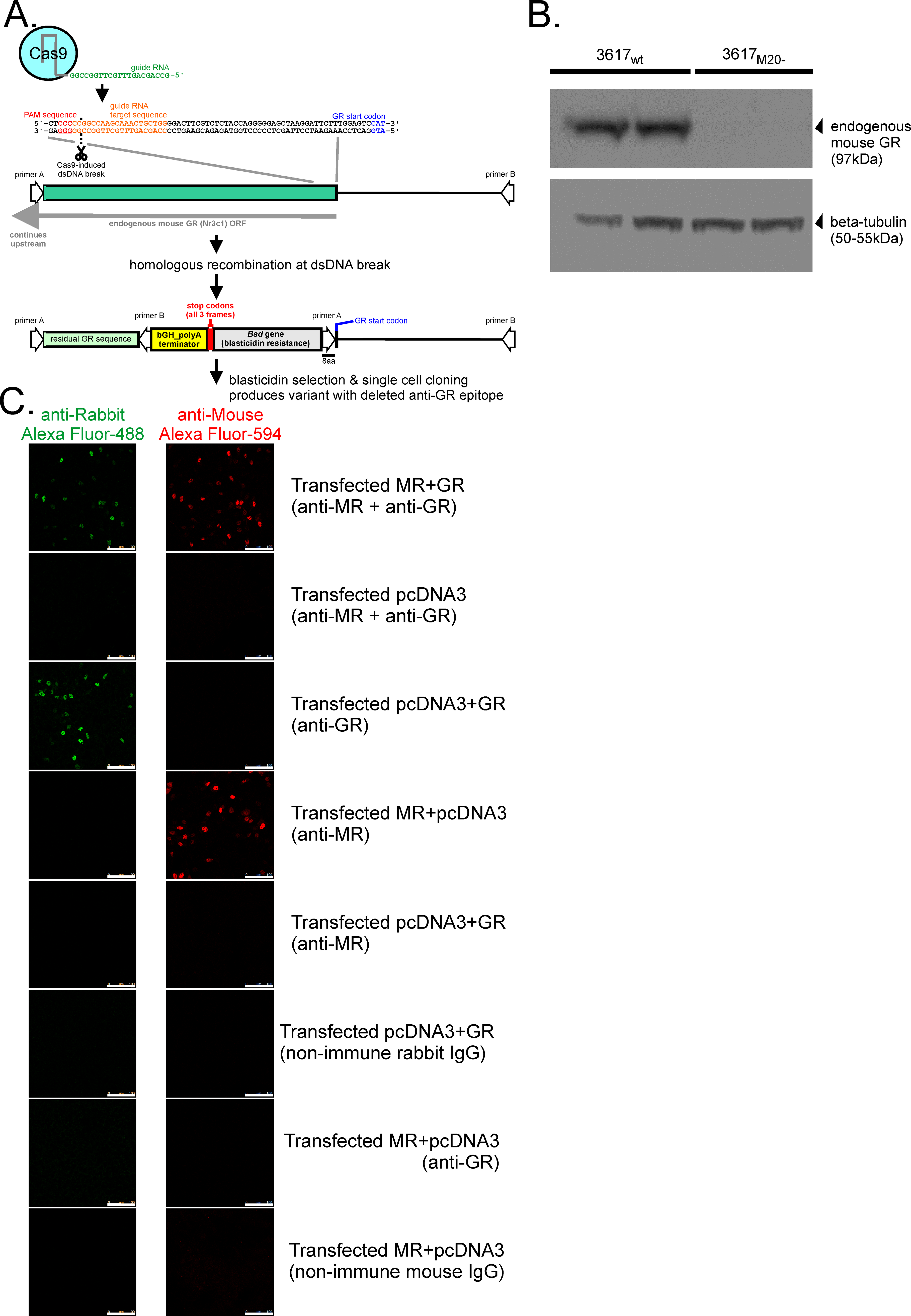

Supplement: S2 Fig — (A) 3617 cells do not express MR but contain endogenous mouse GR. To avoid interference from endogenous GR CRISPR-Cas9 was used to remove the antibody recognition epitope from the first exon of the GR. A guide RNA positions Cas9 close to the start codon of the mouse GR which runs in the antisense direction on chromosome 18, and CRISPR-mediated DNA editing was achieved by homologous recombination between two homology arms one in the GR promoter region and the other positioned toward the end of the GR poly-Q repeat, removing amino acids 3–90 from the protein coding sequence in which the anti-GR antibody epitope lies. The initiating methionine and following aspartic acid were preserved. Deleted sequence was replaced with the blasticidin resistance gene Bsd in frame with the endogenous GR start codon allowing isolation of a monoclonal cell population. (B) Western blot showing the loss of anti-GR M-20 detection of the GR in 3617M20- cells compared to the parental cell line. (C) 3617M20- cells were a negative baseline for immunohistochemistry using the anti-GR M-20 antibody. Cells were transfected +tetracycline with full length rat MR or GR or pcDNA3, corticosterone treated (100 nM, 45 min) and fixed for immunohistochemistry. Primary antibodies were applied as described, all samples received both Alexa Fluor-labelled secondary detection antibodies. MR and GR detection with the primary antibody pair used for PLA was clear and specific demonstrating no cross-reactivity. Scale bar = 100 μm. (TIF) [file pone.0227520.s002.tif]

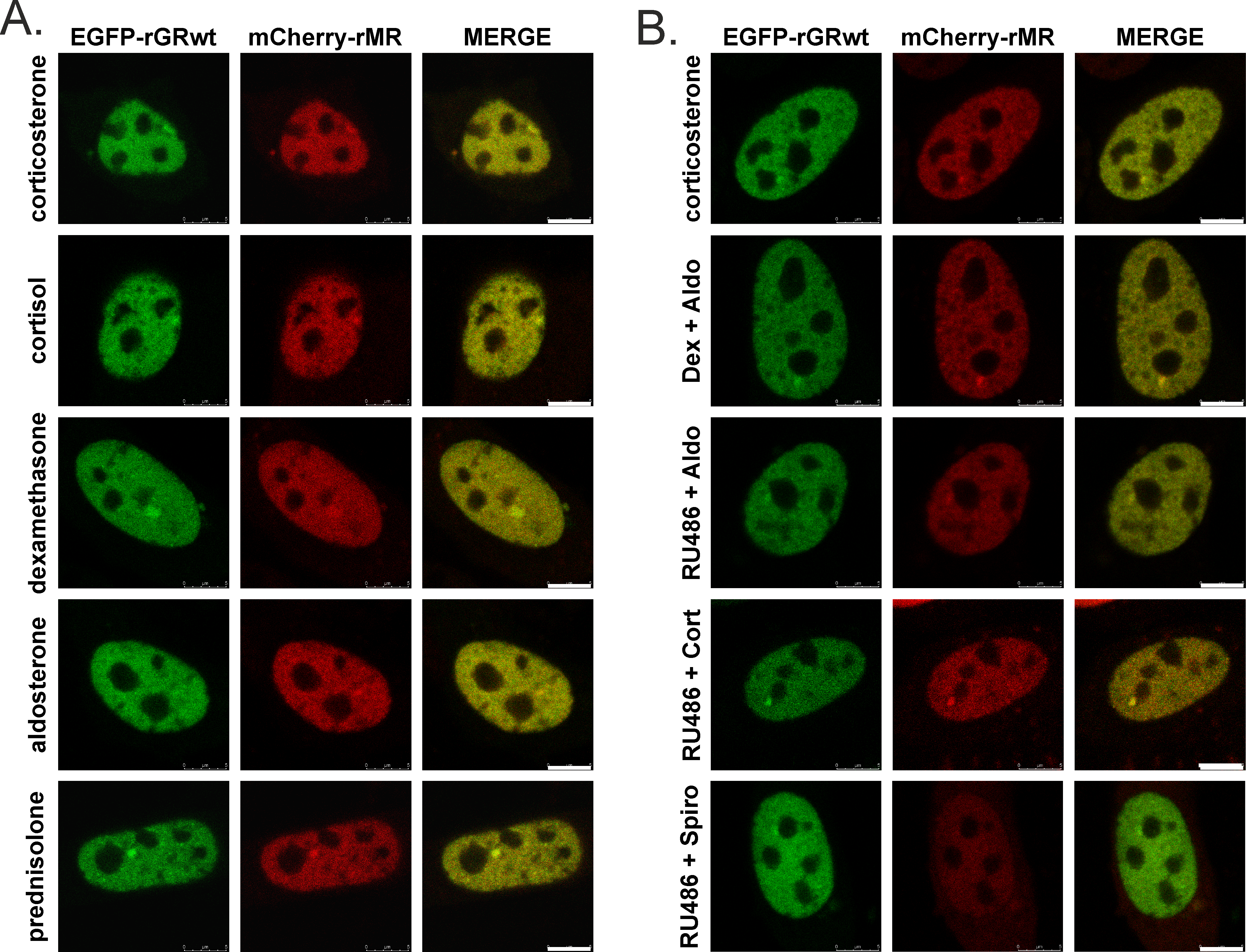

Supplement: S3 Fig — (A) Application of 100 nM of the compounds indicated and compared to corticosterone. (B) Application of combinations of agonists and antagonists. Dexamethasone (Dex) 10 nM + aldosterone (Aldo) 10 nM, spironolactone + RU486 (1 μM each), aldosterone + RU486 and corticosterone + RU486 (10 nM MR-targeted agonist, 1 μM GR-targeted antagonist) were compared to 100 nM corticosterone. Treatments for minimum of 30 min before imaging. Scale bars = 5 μm. (TIF) [file pone.0227520.s003.tif]
