# Supplementary material for: Beyond the heterodimer model for mineralocorticoid and glucocorticoid receptor interactions in nuclei and at DNA
Source: PLoS One. 2020 Jan 10;15(1):e0227520. doi: 10.1371/journal.pone.0227520 (PMC6953809; doi:10.1371/journal.pone.0227520)
Supplement: S2 Table — Predictions are for GR changes and show the average ΔΔG score from alternative mutation analysis software. Colour coding reflects the severity of the change in interaction potential with the darkest blue the strongest predicted change. Note that A477T is the GRdim mutation first demonstrated as a natural mutation in human AR. (DOCX) [file pone.0227520.s005.docx]

| Mutation | BeatMusic | mCSM | MutaBind | FoldX | **Average ΔΔG** |
| --- | --- | --- | --- | --- | --- |
| A477K | 2.59 | 3.48 | 5.10 | 9.46 | 5.16 |
| A477E | 2.53 | 3.79 | 2.94 | 3.31 | 3.14 |
| A477D | 2.06 | 3.86 | 3.04 | 3.24 | 3.05 |
| A477T | 1.05 | 2.23 | 3.30 | 5.22 | 2.95 |
| C482K | 2.26 | 1.13 | 3.57 | 2.65 | 2.40 |
| C482P | 2.07 | 1.16 | 3.64 | 0.46 | 1.83 |
| R488G | 1.90 | 1.40 | 2.31 | 0.40 | 1.50 |
| A477K  C482K |  |  |  | 12.68 | 12.68 |
| A477T  C482K |  |  |  | 8.78 | 8.78 |
| A477K  C482K  R488G |  |  |  | 13.52 | 13.52 |
