# Supplementary material for: Beyond the heterodimer model for mineralocorticoid and glucocorticoid receptor interactions in nuclei and at DNA
Source: PLoS One. 2020 Jan 10;15(1):e0227520. doi: 10.1371/journal.pone.0227520 (PMC6953809; doi:10.1371/journal.pone.0227520)

Original images for co-IP western blot shown in Fig 2A.

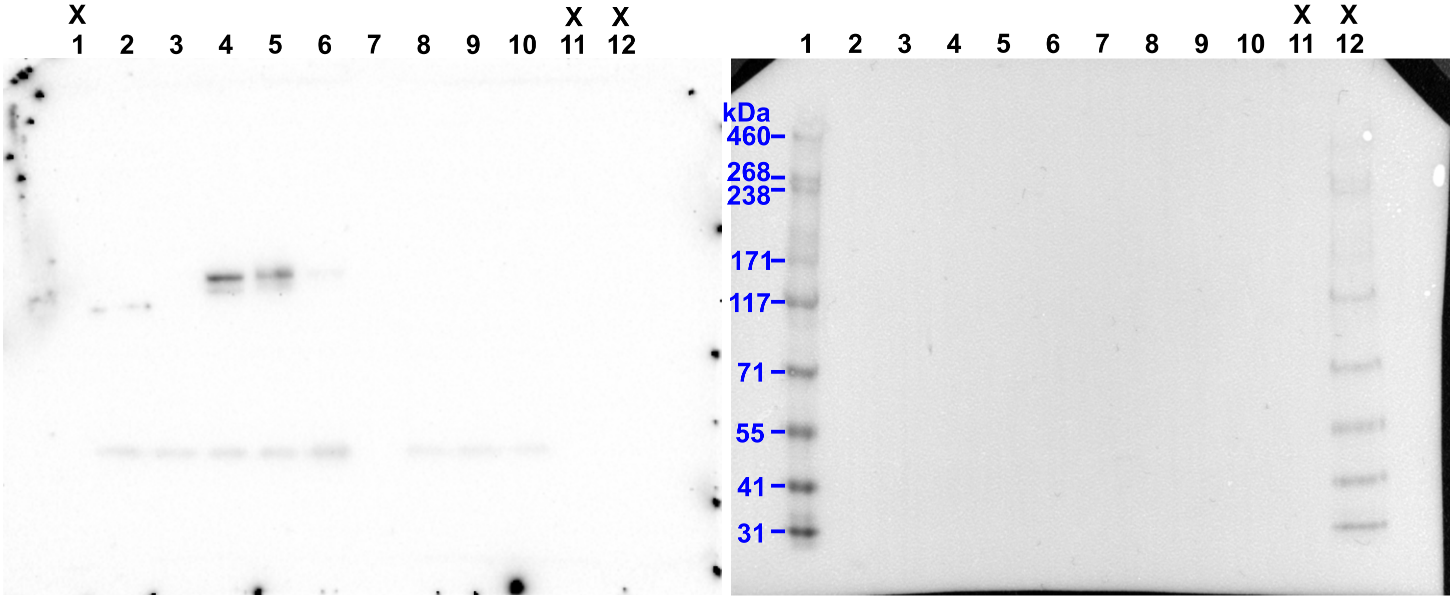

1. Marker
2. Mock IP
3. 3617ChMR
4. 3617ChMR
5. 3617ChMR
6. 3617Ch
7. Blank lane
8. 3617
9. 3617
10. 3617
11. Blank lane
12. Marker

X. Lane not used in Figure

Original images for co-IP western blot shown in Fig 2B.

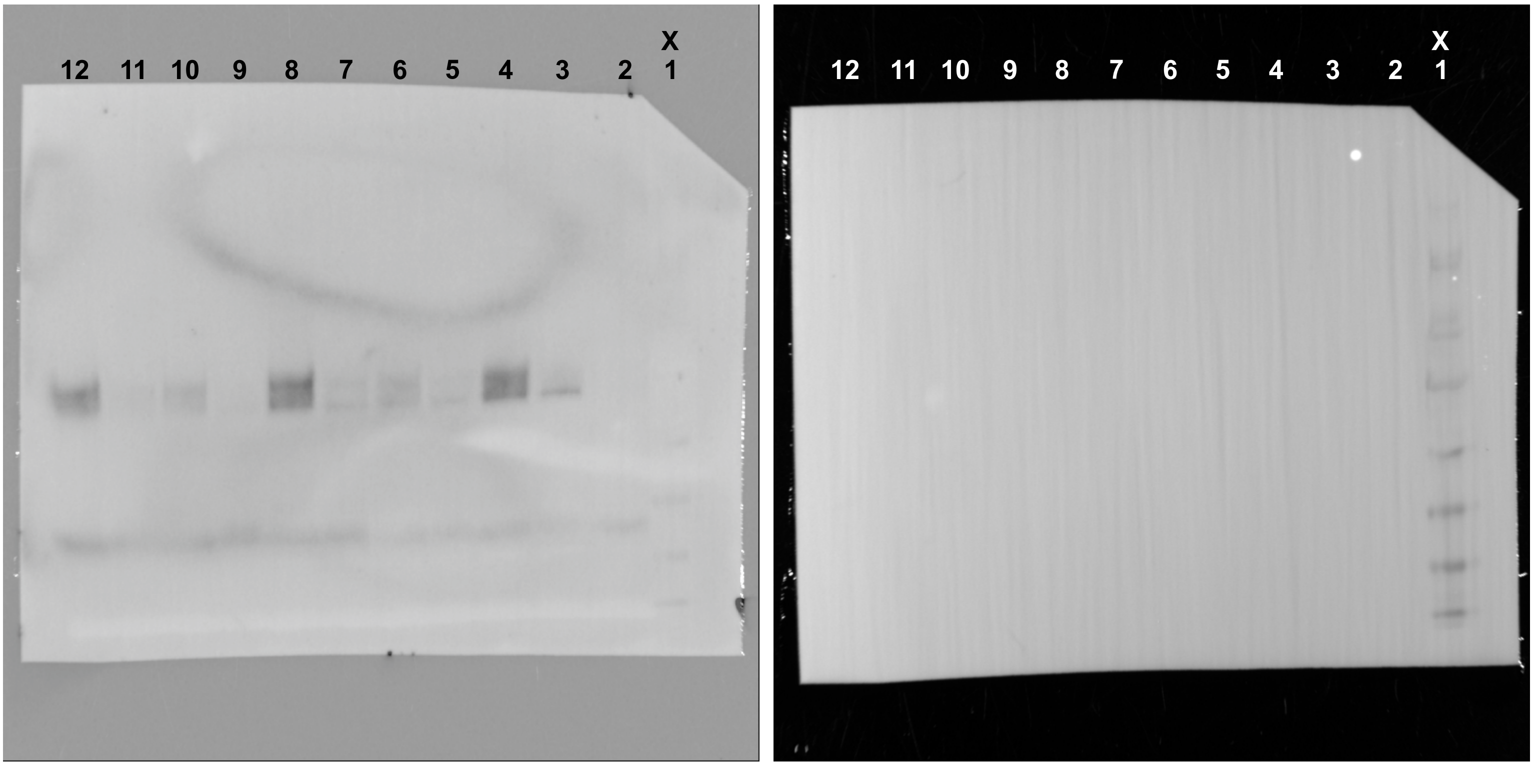

1. Marker
2. Mock IP
3. Rat 3931 -
4. Rat 3931 +
5. Rat 3932 -
6. Rat 3932 +
7. Rat 3933 -
8. Rat 3933 +
9. Rat 3934 -
10. Rat 3934 +
11. Rat 3197 -
12. Rat 3197 +

X. Lane not used in Figure

Original images for co-IP western blot shown in Fig 2C.

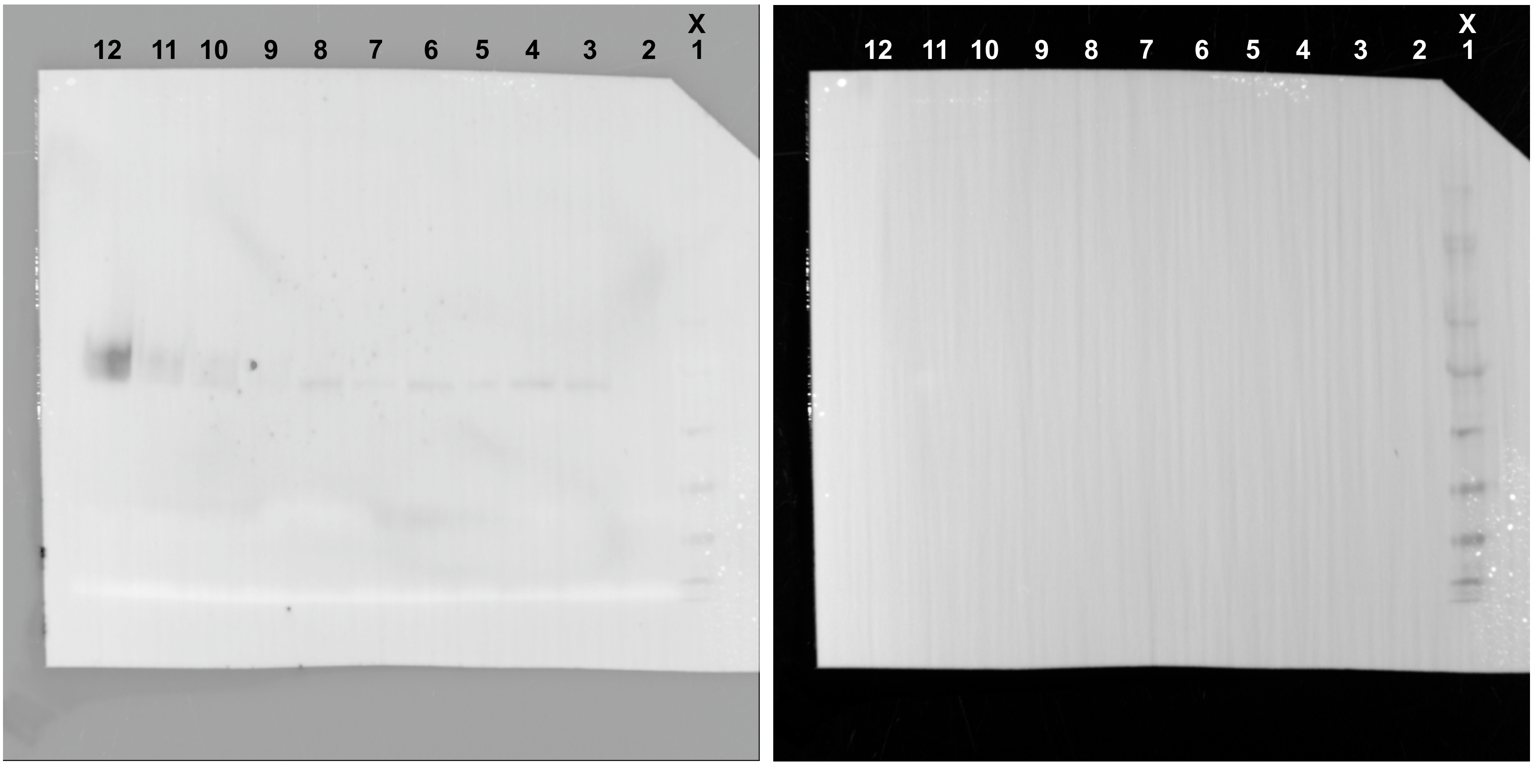

1. Marker
2. Mock IP
3. Rat 3923 -
4. Rat 3923 +
5. Rat 3924 -
6. Rat 3924 +
7. Rat 3925 -
8. Rat 3925 +
9. rat 3926 -
10. Rat 3926 +
11. Rat 3678 -
12. Rat 3678 +

X. Lane not used in Figure

Original images for co-IP western blot shown in Fig 2D.

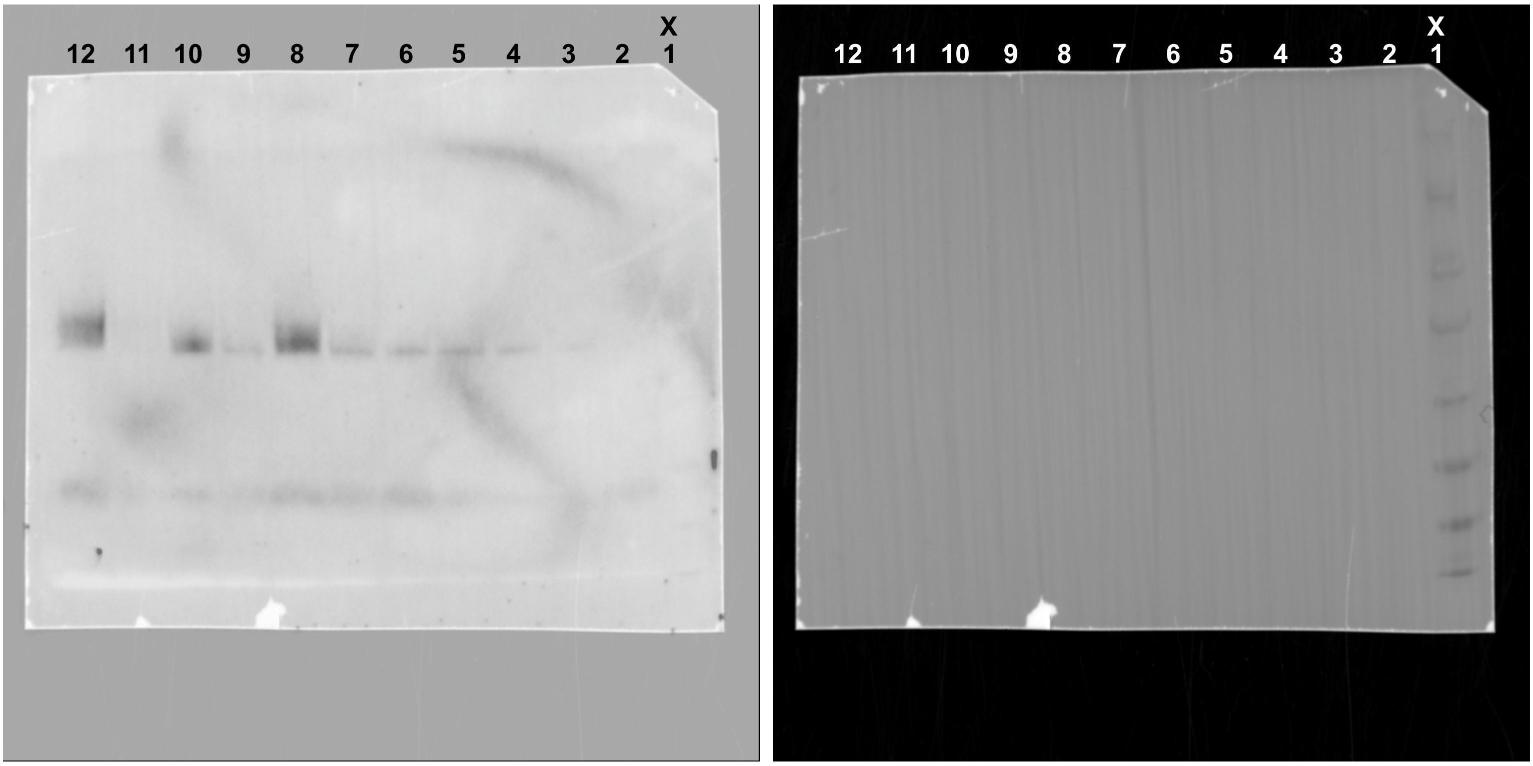

1. Marker
2. Mock IP
3. Rat 4176 -
4. Rat 4176 +
5. Rat 4177 -
6. Rat 4177 +
7. Rat 4123 -
8. Rat 4123 +
9. rat 4125 -
10. Rat 4125 +
11. Rat 3927 -
12. Rat 3927 +

X. Lane not used in Figure

In most cases G:Box composite images did not adequately demonstrate the upper molecular weight markers. Marker positioning was achieved by alignment of the marker image with the composite.

Original images for co-IP western blot shown in S2 Fig B. (two exposures)

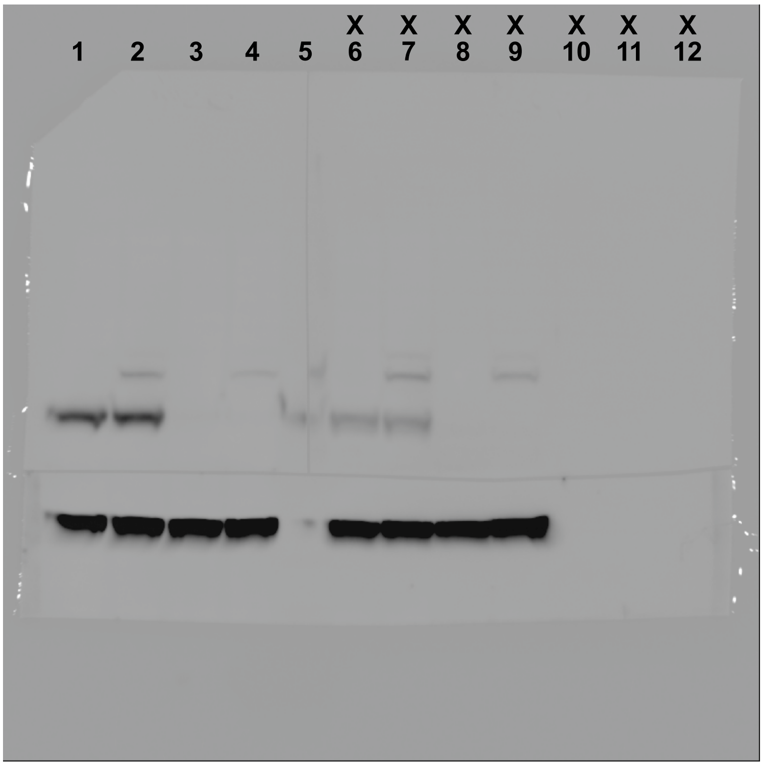

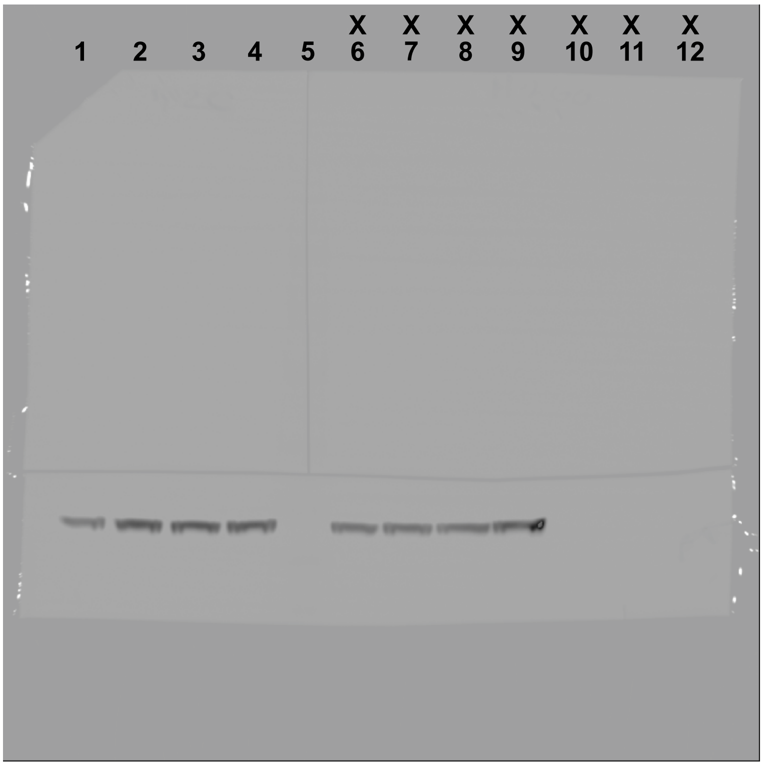

1. 3617wt (with tet)
2. 3617wt (no tet)
3. 3617M20- (with tet)
4. 3617M20- (no tet)
5. Marker

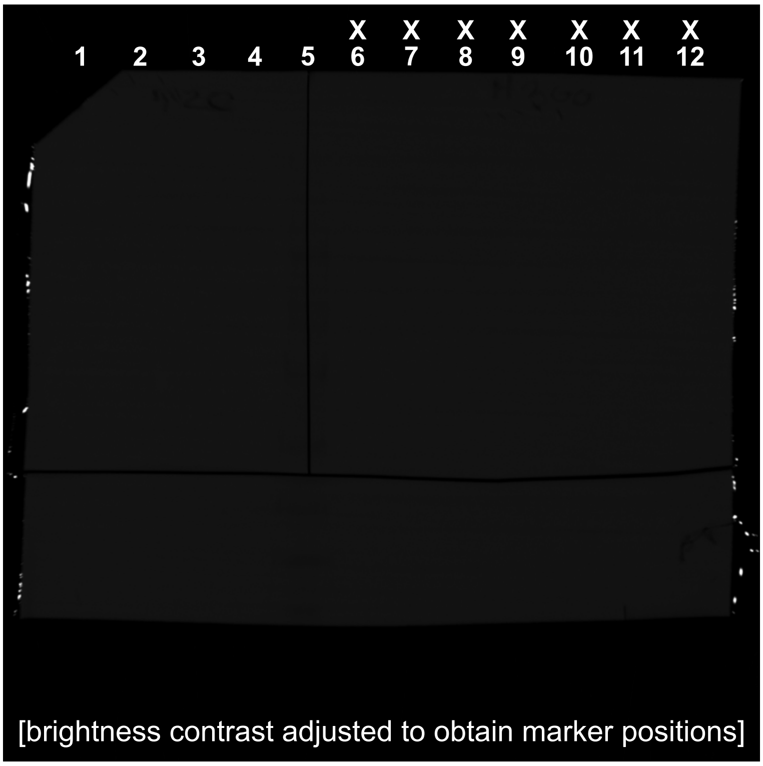

Supplement: S1 Raw Images — (PDF) [file pone.0227520.s009.pdf]
